# Metabolic Dysfunction-Associated Steatotic Liver Disease (MASLD) and Risk of Gynecologic Cancer: A Nationwide Cohort Study

**DOI:** 10.3390/cancers18060894

**Published:** 2026-03-10

**Authors:** Min Jin Jeong, Yong Seok Lee, Youn Jin Choi, Kyung Do Han

**Affiliations:** 1 Department of Obstetrics and Gynecology, Eunpyeong St. Mary’s Hospital, College of Medicine, The Catholic University of Korea, Seoul 03312, Republic of Korea; mjobgy@catholic.ac.kr (M.J.J.);; 2 Department of Obstetrics and Gynecology, Seoul St. Mary’s Hospital, The Catholic University of Korea, 222 Banpo-daero, Seochogu, Seoul 06591, Republic of Korea; 3 Department of Statistics and Actuarial Science, Soongsil University, Seoul 06978, Republic of Korea

**Keywords:** metabolic dysfunction-associated steatotic liver disease, gynecologic neoplasms, cervical neoplasms, endometrial neoplasms, ovarian neoplasms, menopause, cohort studies

## Abstract

Background: Metabolic dysfunction-associated steatotic liver disease (MASLD) is now widely identified as a multisystem disorder with oncogenic implications that extend beyond liver-specific outcomes. Nonetheless, the link between MASLD and gynecologic cancers remains insufficiently characterized in robust, well-powered population studies. We investigated this association by menopausal status in a large cohort of Korean women. Methods: We performed a longitudinal cohort study utilizing data from a nationwide Korean cohort of over 2 million women, with a median observation period of 12.3 years. MASLD, including its subtypes metabolic alcohol-associated liver disease (MetALD), and alcohol-related liver disease (ALD) with metabolic dysfunction were identified using the most recent diagnostic standards. Adjusted hazard ratios (aHR) for gynecologic cancers were estimated with Cox models, accounting for metabolic, reproductive, and lifestyle factors. Results: In premenopausal women, MASLD was associated with increased risks of cervical (aHR, 1.13, 95% CI, 1.01–1.26), endometrial (aHR, 1.63, 95% CI, 1.50–1.79) and ovarian cancer (aHR, 1.22, 95% CI, 1.12–1.33). In postmenopausal women, MASLD similarly conferred elevated risks across all three cancers: cervical (aHR, 1.12, 95% CI, 1.05–1.20), endometrial (aHR, 1.42, 95% CI, 1.32–1.54) and ovarian cancer (aHR, 1.14, 95% CI, 1.08–1.20). Conclusions: MASLD should be considered an independent and modifiable risk determinant for gynecologic cancers. These data underscore the necessity of including hepatic steatosis in risk assessment protocols for cancer prevention. Early recognition and directed screening among metabolically susceptible women may provide important avenues for proactive cancer risk reduction.

## Simple Summary

Metabolic dysfunction-associated steatotic liver disease (MASLD) is increasingly recognized as a systemic condition that influences cancer development beyond the liver. While its impact on various metabolic disorders is well-documented, the specific relationship between MASLD and gynecologic cancers has not been fully explored in large-scale populations. In this study, we demonstrated that MASLD significantly increases the risk of cervical, endometrial, and ovarian cancers regardless of menopausal status, using a comprehensive nationwide database of over 2 million individuals. Our findings suggest that MASLD serves as a critical, modifiable risk factor for gynecologic malignancies. Consequently, integrating liver health assessments into routine gynecologic cancer screening could provide a proactive strategy for early prevention and risk management in women with metabolic dysfunction.

## 1. Introduction

Metabolic dysfunction-associated steatotic liver disease (MASLD), recently renamed from non-alcoholic fatty liver disease (NAFLD), has become a major global public health issue. In addition to liver-related complications, MASLD is increasingly recognized as a disorder affecting multiple organ systems, with heightened risks of cardiovascular disease, chronic kidney disease, and increased all-cause mortality [1,2,3]. Although there is a greater recognition of MASLD and updates to its diagnostic criteria, the global incidence of the disease continues to rise, especially among people with obesity, type 2 diabetes, and metabolic syndrome [4,5]. Recently, research has increasingly focused on extrahepatic outcomes of MASLD, including its potential role in tumorigenesis [6,7,8,9]. Detecting MASLD patients at heightened risk for cancer could support more tailored surveillance and earlier therapeutic interventions [10,11]. While established metabolic risk factors such as obesity, insulin resistance, and chronic inflammation are known contributors to cancer development, the specific and independent effects of hepatic steatosis—particularly in women—have yet to be fully elucidated [12,13].

Gynecologic cancers, including endometrial, ovarian, and cervical malignancies, continue to constitute major sources of cancer burden among women worldwide [14]. Although reproductive and hormonal determinants (e.g., early menarche, nulliparity, unopposed estrogen exposure) are known to impact gynecologic cancer risk, the contribution of metabolic liver disease in this context has not been comprehensively examined [15,16]. New investigations have begun to assess associations between MASLD and cancers specific to women [17,18,19]. Nevertheless, most existing data stem from studies with notable limitations, including small sample sizes, absence of longitudinal analyses, or a focus limited to a single type of gynecologic cancer. Population-based research in Asian women, particularly with stratification by menopausal status, remains sparse.

To address these research gaps, this study explored the association between MASLD and the incidence of endometrial, ovarian, and cervical cancers in a nationwide cohort of over 2 million Korean women. By leveraging an extensive, longitudinal database, we sought to clarify the potential role of MASLD in the pathogenesis of gynecologic cancers across menopausal stages and to provide evidence for guiding screening and preventive measures in metabolically susceptible female populations.

## 2. Materials and Methods

### 2.1. Data Source and Study Population

This retrospective cohort study analyzed data from the Korean National Health Insurance Service (NHIS), a universal, single-payer system that insures approximately 97% of the South Korean population. The NHIS database contains comprehensive longitudinal data, including demographics, International Classification of Diseases, 10th Revision (ICD-10) coded diagnoses, prescription records, healthcare utilization, and results from biennial health screenings [20,21].

Within the National Health Screening Program (NHSP), participants complete standardized assessments every two years that include anthropometric evaluations, laboratory measurements, and validated self-administered questionnaires on lifestyle factors and reproductive history. These datasets have been extensively applied in epidemiologic studies and have contributed to the development of health policy in Korea [22,23].

An initial cohort of 3,109,491 women aged ≥ 40 years who participated in health screening in 2009 were considered for inclusion. Exclusion criteria were incomplete reproductive health questionnaires (n = 383,571), prior hysterectomy (n = 204,288), implausible or missing values related to age at menarche or menopause (n = 181,064), prior cancer diagnosis (n = 56,233), and missing essential covariates (n = 106,752). To reduce the impact of reverse causality, women who died within the first year of follow-up or had follow-up less than 1 year (n = 5761) were also excluded.

Additional exclusions were made for women with a history of liver transplantation (n = 76), concomitant liver diseases (n = 80,949), or those lacking evidence of hepatic steatosis (n = 2499). Concomitant liver diseases included drug-induced liver disease (ICD-10: K71), viral hepatitis (ICD-10: B15-19), hepatic veno-occlusive disease (ICD-10: I82), liver abscess (ICD-10: K75.0; A06.4), hemochromatosis (ICD-10: E83.1), Wilson’s disease (ICD-10: E83.0), alpha-1 antitrypsin deficiency (ICD-10: E88.0), autoimmune hepatitis (ICD-10: K75.4), primary biliary cholangitis (ICD-10: K74.3; K74.4), other cholangitis (ICD-10: K83; K83.0 A), and glycogen storage disease (ICD-10: E74).

The final analytic cohort comprised 2,088,298 women, with follow-up concluding on 31 December 2022. Participants were categorized as premenopausal or postmenopausal based on questionnaire responses. To determine menopausal status in detail, a dedicated section of the questionnaire for female participants asked respondents to indicate their current menstrual status with three options: still experiencing regular periods, having undergone a hysterectomy, or postmenopausal. Participants who reported being postmenopausal were further asked to specify the age at menopause. This structured approach allowed for consistent and reproducible classification of menopausal status across all study subjects.

The cohort had a median follow-up of 12.35 years. Incident gynecologic cancers were identified using ICD-10 codes: cervical cancer (C53), endometrial cancer (C54), and ovarian cancer (C56). Other gynecologic cancers, such as vulvar or vaginal cancers, were not included because the number of cases in our dataset was too small to ensure reliable statistical analysis. Focusing on these three cancer types allowed for meaningful comparative analyses while maintaining the validity and robustness of the study findings.

The study was approved by the Institutional Review Board of the Catholic Medical College of Korea (approval number: PC25ZASI0058). As only de-identified secondary data from the NHIS were utilized, the requirement for informed consent was waived.

### 2.2. Classification of Study Population

In this study, hepatic steatosis was evaluated using the Fatty Liver Index (FLI), which is a well validated, non-invasive surrogate marker. The FLI is a composite score calculated from triglycerides (TG), γ-glutamyl transferase (GGT), body mass index (BMI), and waist circumference (WC). It has demonstrated strong applicability in large-scale epidemiological studies, where imaging and biopsy are often unfeasible because of their invasiveness and associated costs [24]. The FLI was calculated as follows:

$$FLI=\frac{\left[e^{0.953\times ln(TG)+0.139\times BMI+0.718\times ln(GGT)+0.053\times WC-15.745}\right]}{\left[1+e^{0.953\times ln(TG)+0.139\times BMI+0.718\times ln(GGT)+0.053\times WC-15.745}\right]}\times 100$$

The FLI ranges from 0 to 100 and is commonly applied using threshold values: <30 to exclude and ≥60 to confirm hepatic steatosis. Recent studies indicate that, among Asian populations, an FLI ≥ 30 may serve as a more accurate diagnostic benchmark [25,26]. Accordingly, Participants were categorized into the steatotic liver disease (SLD) group (FLI ≥ 30) or the non-SLD group (FLI < 30).

For those identified with SLD, cardiometabolic dysfunction was further assessed and defined by meeting at least one of the following five criteria [27,28,29]: BMI ≥ 23 kg/m^2^ or WC ≥ 85 cm; fasting glucose ≥ 100 mg/dL, a diagnosis of type 2 diabetes, or use of antidiabetic medication; blood pressure ≥ 130/85 mmHg or use of antihypertensive medication; triglycerides ≥ 150 mg/dL or use of lipid-lowering treatment; and HDL-C ≤ 50 mg/dL for women or the use of lipid-lowering treatment.

Furthermore, participants with SLD were stratified based on both metabolic status and alcohol consumption [30]. MASLD was defined as having an FLI ≥ 30, at least one cardiometabolic risk factor, and mild alcohol intake (<20 g/day for women). Participants meeting the MASLD criteria were further classified as MetALD if they reported moderate alcohol intake (20–50 g/day), or as ALD if they reported heavy alcohol intake (≥50 g/day) or possessed any alcohol-related diagnostic code regardless of their intake level.

Participants with SLD but no cardiometabolic risk factors were categorized as non-metabolic SLD and were excluded from the analysis. Furthermore, individuals who otherwise fulfilled the criteria for MASLD but had a prior diagnosis of alcohol abuse (identified using ICD-10 codes: E24.4; F10; G31.2; G62.1; G72.1; I42.6; K29.2; K70; K86.0; Q35.4; R78.0; T51.0; T51.8; T51.9; X65; Y15; Y57.3; Y90; Y91; Z50.2; Z71.4; Z71.2) were excluded from the MASLD group in order to maintain definitional consistency.

### 2.3. Covariates

We obtained detailed information on participants’ demographics (age, income level), lifestyle factors (smoking status, alcohol consumption, physical activity), reproductive history (menopause, parity, breastfeeding, hormone replacement therapy, use of oral contraceptives), anthropometric parameters (height, weight, waist circumference), and blood pressure measurements.

Blood samples were collected after an overnight fast and analyzed for a comprehensive range of biochemical markers, including fasting glucose, total cholesterol, high-density lipoprotein cholesterol (HDL-C), low-density lipoprotein cholesterol (LDL-C), triglycerides (TG), alanine aminotransferase (ALT), aspartate aminotransferase (AST), gamma-glutamyl transferase (GGT), and estimated glomerular filtration rate (eGFR). Body mass index (BMI) was determined by dividing weight in kilograms by height in meters squared (kg/m^2^).

Smoking status was divided into never, former, or current smoker categories. Alcohol consumption was classified as non-drinker, moderate drinker (<20 g/day), or heavy drinker (≥20 g/day). Regular physical activity was defined as engaging in moderate-intensity exercise on ≥5 days per week or vigorous-intensity exercise on ≥3 days per week. Low income was determined as being in the lowest quartile of monthly health insurance premiums or receiving medical aid.

Comorbidities were determined based on diagnostic codes and laboratory data. Diabetes mellitus (DM) was defined by the presence of a diabetes diagnostic code (ICD-10: E11–E14) with concurrent antidiabetic medication use or a fasting glucose level ≥ 126 mg/dL. HbA1c and 2 h post-load glucose were excluded from consideration, as these parameters were not available in the health check-up dataset. Hypertension (HBP) was identified by the presence of a hypertension diagnosis (ICD-10: I10–I13, I15) with antihypertensive treatment or measured blood pressure ≥ 140/90 mmHg. Dyslipidemia was defined as the presence of a dyslipidemia diagnostic code (ICD-10: E78) with lipid-lowering therapy or a total cholesterol concentration ≥ 240 mg/dL.

### 2.4. Statistical Analysis

Descriptive statistics summarized the baseline characteristics of the study population. Continuous variables were reported as means with standard deviations, while categorical variables were presented as frequencies and percentages. Between-group comparisons were performed using one-way analysis of variance for continuous variables and chi-square tests for categorical variables. The normality of continuous variables was assessed through visual inspection of histograms and Q-Q plots, as formal normality tests may be overly sensitive to the large sample size of this study. For skewed variables, including triglycerides, GGT, ALT, and AST, data are presented as median (interquartile range, IQR) and were compared using the Kruskal–Wallis test. Incidence rates of gynecologic cancers—including cervical, endometrial, and ovarian cancers—were determined per 1000 person-years by dividing the number of new cases by the total duration of follow-up. Kaplan–Meier survival curves were generated to depict cumulative incidence across study groups, with group differences evaluated using the log-rank test.

Cox proportional hazards regression models estimated hazard ratios and 95% confidence intervals for associations between steatotic liver disease categories and gynecologic cancer incidence. Multivariable models were constructed in three sequential stages. Model 1 served as an unadjusted, crude reference. Model 2 adjusted for age alone. Model 3 incorporated additional reproductive, behavioral, and socioeconomic covariates, with variables selected according to menopausal status. For premenopausal women, adjustments included factors such as household income, smoking status, regular physical activity, parity, breastfeeding history, history of oral contraceptive (OC) use, and age at menarche. For postmenopausal women, age at menopause and hormone replacement therapy (HRT) were also included. Selection of these covariates was informed by robust epidemiological evidence linking lifestyle and reproductive characteristics to gynecologic cancer risk [31,32,33,34]. Wald test *p*-values for all covariates are provided in Supplementary Table S1. The potential for multicollinearity between BMI and FLI was evaluated prior to the multivariable analysis. Due to the substantial correlation observed between the two variables (*r* = 0.754, *p* < 0.001), BMI was excluded from the final adjusted models to ensure the stability and reliability of the statistical estimates. Statistical analyses were performed using Statistical Analysis System (SAS) version 9.4 (SAS Institute Inc., Cary, NC, USA), with a 2-sided *p* value < 0.05 denoting statistical significance.

## 3. Results

### 3.1. Baseline Characteristics

**Figure 1:**
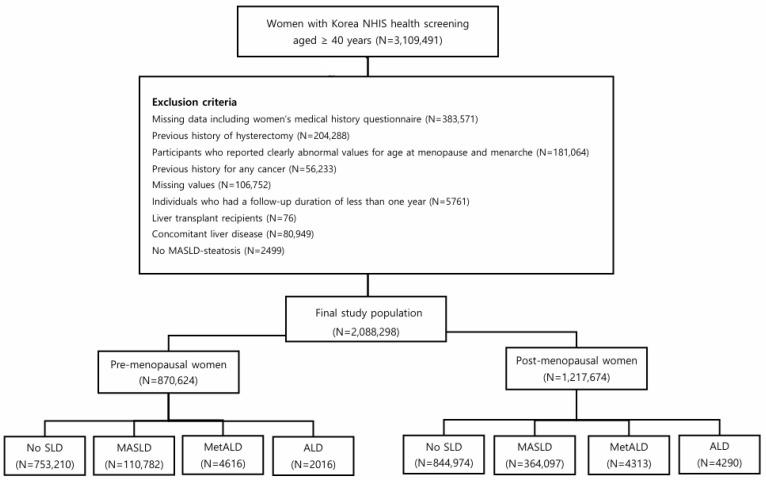
A flow chart of the study population.

This investigation assessed baseline characteristics in a cohort of 2,088,298 women, stratified by menopausal status and by SLD subtypes, including MASLD, MetALD, and ALD. Among 870,624 premenopausal women, participants were assigned to the no SLD group (N = 753,210, 86.51%), MASLD group (N = 110,782, 12.72%), MetALD group (N = 4616, 0.53%), and ALD group (N = 2016, 0.23%). Likewise, 1,217,674 postmenopausal women were distributed into the no SLD group (N = 844,974, 69.39%), MASLD group (N = 364,097, 29.90%), MetALD group (N = 4313, 0.35%), and ALD group (N = 4290, 0.35%), as illustrated in (Figure 1).

Significant differences (*p* < 0.0001) were detected among SLD subtypes for all variables evaluated encompassing demographic and lifestyle characteristics, reproductive history, and both metabolic and hepatic profiles. (See Table 1 for detailed values).

Premenopausal women with MASLD, MetALD, or ALD were older and demonstrated higher BMI, systolic blood pressure, and fasting glucose levels compared to those without SLD. The steatotic groups exhibited more atherogenic lipid profiles, with MASLD presenting the lowest HDL cholesterol and highest LDL cholesterol, while MetALD showed the most elevated triglyceride levels. Prevalence of current smoking was highest in the MetALD and ALD groups (28.1% and 27.3%, respectively). All SLD subtypes were associated with markedly increased liver enzyme levels and FLI, reflecting more advanced hepatic steatosis, as well as higher Charlson Comorbidity Index (CCI) scores. Reproductive cancer–related factors, such as early menarche, nulliparity, and lack of breastfeeding were observed more frequently in the MASLD and especially the MetALD groups compared to those without SLD.

In postmenopausal women, steatotic liver phenotypes continued to be linked to unfavorable metabolic parameters, which paralleled the patterns seen in premenopausal women but with generally higher values. Compared to those without SLD, those with MASLD experienced menopause at a slightly older age and had a longer total reproductive period. The MetALD and ALD groups more commonly exhibited early menopause and shorter reproductive lifespan. HRT use was reported less often among the MASLD group than in other subtypes (Table 2).

### 3.2. Association Between MASLD and Gynecologic Cancer Risk: Comparison Across SLD Subtypes

During a median follow-up of 12.35 years, 22,635 participants (1.08%) received new diagnoses of gynecologic cancers. Among premenopausal and postmenopausal women, there were 6460 cases of cervical cancer (0.26 IR per 1000), 5931 cases of endometrial cancer (0.24 IR per 1000), and 10,244 cases of ovarian cancer (0.41 IR per 1000).

Among premenopausal women, MASLD was independently associated with higher risks for all three gynecologic cancers following full adjustment (Model 3). The highest adjusted hazard ratio for cervical cancer was observed in MetALD (aHR, 1.87, 95% CI, 1.29–2.73), whereas endometrial cancer showed the highest point estimate in MASLD (aHR, 1.63, 95% CI, 1.50–1.79). Ovarian cancer demonstrated numerically elevated aHRs in ALD and MASLD, although these associations did not consistently reach statistical significance. These patterns suggest heterogeneity in risk according to both SLD subtype and cancer type in premenopausal women. Among postmenopausal women, MASLD was consistently associated with increased risks across all gynecologic cancer types. The strongest association was observed for endometrial cancer (aHR, 1.42, 95% CI, 1.32–1.54), followed by cervical and ovarian cancers (aHR, 1.12, 95% CI, 1.05–1.20; aHR, 1.14, 95% CI, 1.08–1.20 for each). In this group, neither MetALD nor ALD showed significant elevations in gynecologic cancer risk (Table 3).

Significant interaction between menopausal status and MASLD was observed for endometrial cancer (*p* for interaction = 0.0001), indicating that the association differed by menopausal status. For cervical and ovarian cancers, no significant interaction was detected (*p* = 0.783, *p* = 0.473, respectively). Overall analyses in the entire population showed results consistent with the stratified analyses: MASLD was associated with an increased risk of cervical (aHR, 1.12, 95% CI, 1.06–1.19), endometrial (aHR 1.50, 95% CI, 1.42–1.60) and ovarian cancer (aHR 1.16, 95% CI, 1.10–1.21). (Supplementary Table S2). These findings indicate that, while menopausal status modifies the association for endometrial cancer, the overall association of MASLD with gynecologic cancer risk remains consistent across the population.

**Figure 2:**
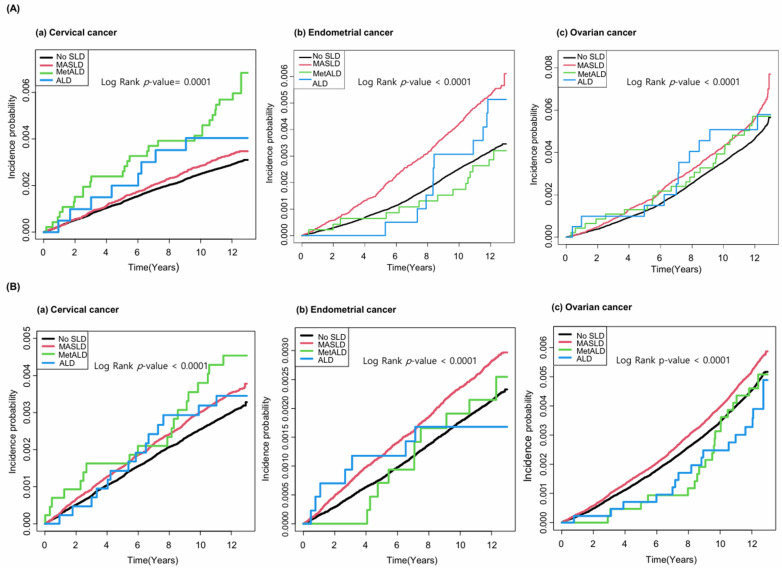
Linear association between MASLD and increased gynecologic cancer risk in (**A**) premenopausal and (**B**) postmenopausal women.

Kaplan–Meier survival analysis further showed that women with MASLD had a higher cumulative incidence of cervical, endometrial, and ovarian cancers compared to those without steatosis, irrespective of menopausal status (Figure 2). All observed differences reached statistical significance (log-rank *p* < 0.0001).

### 3.3. Subgroup Analysis

We further assessed the associations between SLD subtypes and gynecologic cancer risk according to menopausal status as well as reproductive and lifestyle factors (Supplementary Table S3). Notably, significant effect modifications were identified for parity and breastfeeding history, especially in relation to endometrial cancer. Among premenopausal women, MASLD was linked to a substantially higher risk of endometrial cancer in those without a history of parity or breastfeeding. Absence of regular physical activity correlated with heightened risk of all three gynecologic cancers across both menopausal groups. Despite this, interaction effects for physical activity were not statistically significant. Hormone-related factors, including hormone replacement therapy and oral contraceptive use, were associated with varying cancer risks depending on type. Additional factors, including smoking, age at menarche, and age at menopause, did not significantly alter the link between SLD and gynecologic cancer risk.

To address the possible impact of menopausal transition occurring during follow-up, we performed a sensitivity analysis by restricting the follow-up period to age 55 years. The results were unchanged, supporting the reliability of our main findings (Supplementary Table S4).

### 3.4. Sensitivity Analysis

To evaluate the robustness of our findings according to the severity of hepatic steatosis, a sensitivity analysis was performed using a higher FLI cutoff of 60 (Supplementary Table S5). The overall results were largely consistent with the primary analysis (FLI ≥ 30). Notably, while the association between MetALD and cervical cancer was statistically significant only in premenopausal women in the primary analysis, it reached statistical significance in postmenopausal women as well when the more stringent FLI 60 threshold was applied (aHR, 2.22, 95% CI, 1.19–4.14).

## 4. Discussion

This large nationwide cohort study of over two million women demonstrates compelling epidemiological evidence that MASLD is linked with an increased risk of gynecologic cancers, including cervical, endometrial, and ovarian cancer. These associations were observed consistently across menopausal strata and remained robust after comprehensive adjustment for metabolic, reproductive, and lifestyle risk factors. Notably, the significant associations found in premenopausal women provide crucial evidence for the metabolic origins of early-onset gynecologic cancers. Our findings build upon previous smaller studies by utilizing a large, population-based cohort with extended follow-up and by implementing current MASLD diagnostic criteria in line with recent consensus [11,35]. Importantly, we implemented the recently introduced nomenclature that distinguishes MASLD, MetALD, and ALD. According to this system, MASLD requires hepatic steatosis plus at least one cardiometabolic risk factor in the absence of significant alcohol consumption. MetALD identifies individuals matching MASLD criteria but also engaging in moderate alcohol use, thus indicating combined effects of metabolic dysfunction and alcohol. ALD designates steatosis that results predominantly from high alcohol consumption, regardless of metabolic background. This updated classification, supported by the 2023 international consensus [36], more precisely differentiates metabolic from alcohol-induced pathways, enabling improved study comparability and increasing the clinical and public health relevance of epidemiologic results, particularly in understanding how these distinct pathways contribute to cancer risk in younger versus older populations.

Unlike prior studies that were often hindered by small sample sizes, brief follow-up, or inadequate adjustment for confounding, our research simultaneously assessed several gynecologic cancer types within a nationwide cohort and stratified participants by menopausal status. This approach yields new perspectives on MASLD risk patterns across female age groups, highlighting that metabolic dysfunction is a potent driver of malignancy even in the early-onset demographic, as represented by our premenopausal cohort. Although Our results align with existing evidence from meta-analyses and studies such as the UK Biobank [37,38,39], we further demonstrate these associations in a large Asian sample with enhanced statistical reliability and mor comprehensive adjustment for reproductive and behavior factors [40,41].

In this study, MASLD was associated with an increased risk of gynecologic cancers in both premenopausal and postmenopausal women, with the strongest and most consistent association observed for endometrial cancer. Specifically, endometrial cancer showed a high point estimate in MASLD for premenopausal women (aHR, 1.63; 95% CI, 1.50–1.79) and remained the strongest association in postmenopausal women (aHR, 1.42; 95% CI, 1.32–1.54). The particularly strong association between MASLD and endometrial cancer may be explained by alterations in sex hormone metabolism. The liver plays a central role in estrogen regulation, and MASLD-related hepatic dysfunction may reduce sex hormone–binding globulin (SHBG) levels, increase peripheral aromatization, and promote hyperinsulinemia [7,16,31]. These metabolic and hormonal changes can enhance unopposed estrogen exposure and endometrial proliferation, providing a biologically plausible mechanism for the elevated risk observed in our findings [42,43].

For cervical cancer, the higher risk estimates observed in MetALD among premenopausal women (aHR, 1.87, 95% CI, 1.29–2.73) may reflect the combined effects of alcohol-related immune modulation and metabolic dysfunction, although this finding should be interpreted cautiously [26,33]. The associations observed for ovarian cancer were more modest (aHR, 1.14 in postmenopausal women) and may involve metabolic and inflammatory pathways rather than direct hormonal mechanisms [41].

We performed interaction tests to determine if the association between MASLD and gynecologic cancer risk varied by menopausal status. Our findings indicate a significant interaction between menopausal status and MASLD in relation to endometrial cancer, with a stronger association observed in specific menopausal groups (*p* for interaction = 0.0001). In contrast, no significant interactions were detected for cervical or ovarian cancers, suggesting that MASLD-associated risk for these cancers is not substantially modified by menopausal status. Overall analyses in the entire study population showed results consistent with the stratified analyses, indicating that MASLD was associated with increased risks of endometrial, ovarian, and cervical cancers (Supplementary Table S2). These findings support a broad role of metabolic liver dysfunction in gynecologic cancer risk, while highlighting that menopausal status specifically modifies the association for endometrial cancer.

Analyses of subgroups indicate a possible interaction between MASLD risk and reproductive or behavioral characteristics (Supplementary Table S6). Specifically, nulliparity, absence of breastfeeding, and lack of physical activity tended to increase MASLD-associated risk, most notably in endometrial cancer. These findings emphasize potential synergistic effects between metabolic disturbance and known hormonal or behavioral risk factors. However, it should be noted that the interaction effects across most subgroups did not reach statistical significance (*p* for interaction > 0.05). This may be attributed to limited statistical power resulting from the relatively small number of incident cases within specific subcategories. Nevertheless, the consistent direction of effect sizes across these strata suggests that the observed associations are reliable, warranting further large-scale epidemiologic and mechanistic studies to confirm these potential synergies.

The robustness of our findings is underscored by the consistency observed in the sensitivity analysis utilizing an FLI threshold of 60 [24]. While the primary analysis primarily identified a heightened risk of cervical cancer among premenopausal women within the MetALD cohort, the emergence of a statistically significant association in the postmenopausal subgroup at this more stringent cutoff is a pivotal observation.

This finding implies that the oncogenic potential of MetALD in postmenopausal individuals may be contingent upon reaching a critical metabolic tipping point. Unlike their premenopausal counterparts, who appear more susceptible to the early stages of metabolic impairment, postmenopausal women may require a more advanced degree of hepatic steatosis and systemic inflammation to significantly escalate the risk of cervical malignancy [32,33]. The fact that this association became evident only at the FLI 60 level suggests that the synergistic interplay between chronic alcohol consumption and severe metabolic derangement may override the physiological distinctions typically imposed by menopausal status [1,12,15]. Consequently, these data categorize severe MetALD as a substantial and universal risk factor for cervical cancer, highlighting the necessity for vigilant clinical screening in patients with high FLI scores, regardless of their hormonal environment.

There are important limitations to consider. First, MASLD was identified through non-invasive surrogate indices instead of histologic confirmation, which may introduce exposure misclassification. Although extensive confounding adjustment was performed, residual confounding by factors such as dietary patterns, genetic predisposition, or cancer screening variability remains possible. Detection bias could also arise if women with metabolic disease had greater medical surveillance, potentially facilitating earlier cancer diagnosis. Additionally, the absence of molecular subtype data restricted our assessment of variation in associations among cancer subtypes. In relation to our diagnostic approach, we utilized the Fatty Liver Index (FLI) cutoff of 30, as it is currently the most widely validated benchmark for Asian populations, ensuring both diagnostic specificity and comparability with existing literature. While our internal observations suggested potential metabolic shifts within the FLI 20–29 range, we prioritized the established threshold to provide a rigorous and standardized analysis. However, this may leave a subtle sensitivity gap in capturing subclinical cases. Future research is warranted to determine if a more granular stratification could improve early detection in Asian women.

Furthermore, while causal inference cannot be definitively established using an observational design, the robustness of associations across analytic methods, their persistence following comprehensive adjustment, and established biological plausibility collectively enhance the argument for a potential causal linkage. Lastly, approximately one-third of the initial participants were excluded from the analysis. Although these exclusions were primarily based on pre-defined study criteria and the requirement for complete exposure and outcome data, they may have introduced potential selection bias. However, given that the exclusions were systematic and not related to the exposures or outcomes of interest, it is unlikely that they substantially affected the observed associations in this study.

The implications for public health are considerable. MASLD is highly prevalent, often asymptomatic, and increasing in incidence globally [44,45,46]. Given the rising global burden of early-onset cancers, our results emphasize the necessity of integrating metabolic liver health into cancer risk assessment and prevention efforts for women of all ages. Timely identification and monitoring of women at increased metabolic risk may allow for personalized interventions that reduce the escalating incidence of gynecologic cancers. Future investigations should corroborate these results in heterogeneous populations, employ mor precise MASLD phenotyping and cancer subtyping, and evaluate gene–environment interactions to clarify pathogenic mechanisms. Prospective studies of emerging metabolic interventions and their effects on gynecologic cancer risk will be crucial for guiding prevention initiatives and clinical recommendations aimed at mitigating the risk of early-onset malignancies.

## 5. Conclusions

In summary, this large-scale cohort study supplies compelling epidemiologic evidence implicating MASLD as an independent metabolic risk factor for gynecologic cancers, particularly in the context of early-onset malignancies among premenopausal women. These results underscore the significance of metabolic liver health throughout the female lifespan as a modifiable determinant for cancer prevention. Our findings suggest that early metabolic intervention may serve as a critical strategy for reducing the rising global burden of early-onset cancers at both clinical and population health levels.

Further longitudinal studies and clinical trials are warranted to elucidate the underlying biological mechanisms and to evaluate the long-term efficacy of specific metabolic interventions in reducing the incidence of gynecologic malignancies. Future research should also focus on identifying the optimal timing and duration of these interventions to maximize their preventive impact across diverse populations.

## Figures and Tables

**Table 1:** Baseline participant characteristics by SLD subtypes.

	In Pre-Menopause						In Post-Menopause					
Characteristics	Total N = 870,624	No SLD N = 753,210	MASLD N = 110,782	MetALD N = 4616	ALD N = 2016	*p*-Value	Total N = 1,217,674	No SLD N = 844,974	MASLD N = 364,097	MetALD N = 4313	ALD N = 4290	*p*-Value
Age (years)	45.08 ± 4.23	44.92 ± 4.1	46.16 ± 4.86	45.53 ± 3.98	46.04 ± 4.33	<1.0 × 10^−13^	61.68 ± 8.44	61.08 ± 8.56	63.12 ± 8	57.64 ± 7.05	61.04 ± 7.94	<1.0 × 10^−13^
Low-income status ^a^	215,926 (24.8)	186,436 (24.75)	27,628 (24.94)	1269 (27.49)	593 (29.41)	9.81 × 10^−9^	262,391 (21.55)	184,988 (21.89)	75,270 (20.67)	1148 (26.62)	985 (22.96)	<1.0 × 10^−13^
Smoking						<1.0 × 10^−13^						<1.0 × 10^−13^
Never Former Current	827,813 (95.08) 13,749 (1.58) 29,062 (3.34)	718,821 (95.43) 11,642 (1.55) 22,747 (3.02)	104,563 (94.39) 1752 (1.58) 4467 (4.03)	3050 (66.07) 268 (5.81) 1298 (28.12)	1379 (68.4) 87 (4.32) 550 (27.28)		1,172,692 (96.31) 12,634 (1.04) 32,348 (2.66)	815,927 (96.56) 8261 (0.98) 20,786 (2.46)	349,886 (96.1) 4064 (1.12) 10,147 (2.79)	3181 (73.75) 210 (4.87) 922 (21.38)	3698 (86.2) 99 (2.31) 493 (11.49)	
Drinking ^b^						<1.0 × 10^−13^						<1.0 × 10^−13^
Non Mild Heavy	623,930 (71.66) 225,103 (25.86) 21,591 (2.48)	541,978 (71.96) 195,354 (25.94) 15,878 (2.11)	81,396 (73.47) 29,386 (26.53) 0 (0)	0 (0) 0 (0) 4616 (100)	556 (27.58) 363 (18.01) 1097 (54.41)		1,067,081 (87.63) 137,789 (11.32) 12,804 (1.05)	740,961 (87.69) 96,564 (11.43) 7449 (0.88)	323,531 (88.86) 40,566 (11.14) 0 (0)	0 (0) 0 (0) 4313 (100)	2589 (60.35) 659 (15.36) 1042 (24.29)	
Regular exercise ^c^	149,984 (17.23)	132,050 (17.53)	16,792 (15.16)	808 (17.5)	334 (16.57)	<1.0 × 10^−13^	222,252 (18.25)	161,781 (19.15)	58,943 (16.19)	834 (19.34)	694 (16.18)	<1.0 × 10^−13^
DM	30,451 (3.5)	16,629 (2.21)	13,078 (11.81)	461 (9.99)	283 (14.04)	<1.0 × 10^−13^	155,261 (12.75)	75,846 (8.98)	77,537 (21.3)	741 (17.18)	1137 (26.5)	<1.0 × 10^−13^
HBP	121,266 (13.93)	82,109 (10.9)	36,574 (33.01)	1708 (37)	875 (43.4)	<1.0 × 10^−13^	555,112 (45.59)	322,462 (38.16)	227,368 (62.45)	2461 (57.06)	2821 (65.76)	<1.0 × 10^−13^
Dyslipidemia	93,373 (10.72)	64,251 (8.53)	27,495 (24.82)	1018 (22.05)	609 (30.21)	<1.0 × 10^−13^	396,740 (32.58)	231,528 (27.4)	161,192 (44.27)	1768 (40.99)	2252 (52.49)	<1.0 × 10^−13^
Age at menarche, years	15.09 ± 1.68	15.07 ± 1.66	15.18 ± 1.8	15.42 ± 1.8	15.47 ± 1.78	<1.0 × 10^−13^	16.46 ± 1.84	16.42 ± 1.85	16.55 ± 1.82	16.47 ± 1.87	16.6 ± 1.87	<1.0 × 10^−13^
Age at menarche						<1.0 × 10^−13^						<1.0 × 10^−13^
≤12 ≤14 ≤16 >16	39,296 (4.51) 274,932 (31.58) 398,570 (45.78) 157,826 (18.13)	33,077 (4.39) 240,195 (31.89) 347,412 (46.12) 132,526 (17.59)	5948 (5.37) 33,066 (29.85) 48,167 (43.48) 23,601 (21.3)	185 (4.01) 1189 (25.76) 2091 (45.3) 1151 (24.94)	86 (4.27) 482 (23.91) 900 (44.64) 548 (27.18)		11,733 (0.96) 147,637 (12.12) 471,657 (38.73) 586,647 (48.18)	8459 (1) 106,119 (12.56) 331,947 (39.28) 398,449 (47.16)	3156 (0.87) 40,525 (11.13) 136,492 (37.49) 183,924 (50.52)	58 (1.34) 548 (12.71) 1632 (37.84) 2075 (48.11)	60 (1.4) 445 (10.37) 1586 (36.97) 2199 (51.26)	
OC						<1.0 × 10^−13^						<1.0 × 10^−13^
Non <1 year ≥1 year	758,214 (87.09) 82,769 (9.51) 29,641 (3.4)	658,270 (87.4) 70,730 (9.39) 24,210 (3.21)	94,928 (85.69) 11,093 (10.01) 4761 (4.3)	3468 (75.13) 675 (14.62) 473 (10.25)	1548 (76.79) 271 (13.44) 197 (9.77)		1,029,728 (84.57) 113,059 (9.28) 74,887 (6.15)	718,133 (84.99) 77,879 (9.22) 48,962 (5.79)	304,684 (83.68) 34,272 (9.41) 25,141 (6.91)	3383 (78.44) 488 (11.31) 442 (10.25)	3528 (82.24) 420 (9.79) 342 (7.97)	
Parity						<1.0 × 10^−13^						<1.0 × 10^−13^
0 1 ≥2	31,633 (3.63) 115,182 (13.23) 723,809 (83.14)	27,851 (3.7) 99,797 (13.25) 625,562 (83.05)	3446 (3.11) 14,257 (12.87) 93,079 (84.02)	218 (4.72) 784 (16.98) 3614 (78.29)	118 (5.85) 344 (17.06) 1554 (77.08)		20,193 (1.66) 71,929 (5.91) 1,125,552 (92.43)	15,267 (1.81) 54,768 (6.48) 774,939 (91.71)	4686 (1.29) 16,407 (4.51) 343,004 (94.21)	130 (3.01) 457 (10.6) 3726 (86.39)	110 (2.56) 297 (6.92) 3883 (90.51)	
Breast Feeding						<1.0 × 10^−13^						<1.0 × 10^−13^
No <6 months 6–12 months ≥12 months	155,576 (17.87) 214,119 (24.59) 229,559 (26.37) 271,370 (31.17)	135,475 (17.99) 191,909 (25.48) 200,971 (26.68) 224,855 (29.85)	18,713 (16.89) 21,001 (18.96) 27,147 (24.5) 43,921 (39.65)	934 (20.23) 872 (18.89) 1007 (21.82) 1803 (39.06)	454 (22.52) 337 (16.72) 434 (21.53) 791 (39.24)		77,322 (6.35) 75,813 (6.23) 206,547 (16.96) 857,992 (70.46)	58,301 (6.9) 59,752 (7.07) 152,879 (18.09) 574,042 (67.94)	18,247 (5.01) 15,608 (4.29) 52,408 (14.39) 277,834 (76.31)	444 (10.29) 267 (6.19) 673 (15.6) 2929 (67.91)	330 (7.69) 186 (4.34) 587 (13.68) 3187 (74.29)	
Height (cm)	157.51 ± 5.16	157.6 ± 5.13	156.91 ± 5.34	157.28 ± 5.2	157.19 ± 5.29	<1.0 × 10^−13^	153.44 ± 5.73	153.56 ± 5.78	153.13 ± 5.6	154.66 ± 5.41	153.59 ± 5.59	<1.0 × 10^−13^
Weight (kg)	57.61 ± 8.08	55.94 ± 6.54	68.44 ± 8.7	66.29 ± 8.82	65.72 ± 9.37	<1.0 × 10^−13^	56.99 ± 8.28	54.12 ± 6.72	63.48 ± 7.81	63.51 ± 8.55	62.93 ± 8.35	<1.0 × 10^−13^
BMI (kg/m^2^)	23.22 ± 3.06	22.51 ± 2.37	27.78 ± 3.12	26.79 ± 3.33	26.59 ± 3.55	<1.0 × 10^−13^	24.18 ± 3.15	22.93 ± 2.36	27.05 ± 2.82	26.53 ± 3.11	26.65 ± 3.05	<1.0 × 10^−13^
Waist circumference (cm)	75.19 ± 7.74	73.46 ± 6.23	86.37 ± 7.18	85.35 ± 7.61	84.99 ± 7.9	<1.0 × 10^−13^	80.07 ± 8.28	76.7 ± 6.42	87.73 ± 6.81	86.47 ± 7.39	86.98 ± 7.35	<1.0 × 10^−13^
SBP (mmHg)	117.28 ± 14.35	115.98 ± 13.74	125.54 ± 15.31	127.22 ± 15.79	126.18 ± 15.55	<1.0 × 10^−13^	125.74 ± 16.19	123.7 ± 15.87	130.38 ± 15.94	130.79 ± 16.42	129.83 ± 16.05	<1.0 × 10^−13^
DBP (mmHg)	73.18 ± 9.96	72.33 ± 9.62	78.52 ± 10.37	80.35 ± 10.87	79.63 ± 10.59	<1.0 × 10^−13^	76.97 ± 10.17	75.88 ± 10.02	79.44 ± 10.05	80.86 ± 10.47	79.76 ± 10.24	<1.0 × 10^−13^
HDL-C (mg/dL)	60 ± 30.07	60.58 ± 26.89	55.95 ± 45.6	62 ± 39.67	60.09 ± 36.64	<1.0 × 10^−13^	57.57 ± 33.33	59 ± 32.13	54.22 ± 35.71	60.85 ± 31.77	57 ± 35.57	<1.0 × 10^−13^
LDL-C (mg/dL)	113.15 ± 35.63	112.2 ± 34.37	119.92 ± 42.27	108.51 ± 39.42	108.1 ± 47.18	<1.0 × 10^−13^	125.76 ± 38.33	125.75 ± 36.68	125.91 ± 41.83	122.61 ± 40.88	119.41 ± 42.2	<1.0 × 10^−13^
Fasting glucose (mg/dL)	93.67 ± 17.85	92.24 ± 15.24	102.75 ± 27.91	103.43 ± 26.31	105.04 ± 28.7	<1.0 × 10^−13^	99.68 ± 24.25	96.86 ± 20.92	106.01 ± 29.48	108.1 ± 29.75	109.03 ± 30.36	<1.0 × 10^−13^
Total cholesterol (mg/dL)	192.05 ± 34	189.61 ± 32.69	207.84 ± 37.83	206.26 ± 36.78	205.12 ± 40.21	<1.0 × 10^−13^	208.33 ± 38.71	205.09 ± 37.17	215.64 ± 41.02	220.58 ± 41.19	212.52 ± 42.98	<1.0 × 10^−13^
eGFR (mL/min/1.73 m^2^) ^d^	91.62 ± 18.18	91.83 ± 18.03	90.16 ± 19.13	92.19 ± 18.1	91.32 ± 19.28	<1.0 × 10^−13^	79.99 ± 17.98	80.86 ± 17.7	77.94 ± 18.44	83.8 ± 17.8	79.52 ± 18.41	<1.0 × 10^−13^
FLI	14.7 ± 16.14	9.34 ± 7	48.84 ± 15.75	52.18 ± 17.35	54.34 ± 17.63	<1.0 × 10^−13^	24.46 ± 19.63	13.51 ± 7.74	49.19 ± 15.27	53.47 ± 17.31	53.71 ± 16.95	<1.0 × 10^−13^
Triglyceride (mg/dL) ^e^	86 (62–121)	80 (60–107)	156 (115–217)	161 (117–227)	166 (121–241)	<1.0 × 10^−13^	115 (82–162)	99 (74–133)	166 (124–227)	163 (119–232)	162 (121–229)	<1.0 × 10^−13^
GGT (IU/L) ^e^	16 (12–22)	15 (12–20)	27 (20–42)	42 (28–70)	49 (29–90)	<1.0 × 10^−13^	19 (14–27)	17 (13–22)	27 (20–40)	44 (29–75)	39 (26–73)	<1.0 × 10^−13^
ALT (IU/L) ^e^	16 (12–21)	15 (12–19)	23 (17–33)	22 (17–31)	25 (18–37)	<1.0 × 10^−13^	19 (15–26)	18 (14–23)	23 (18–32)	24 (18–33)	26 (19–38)	<1.0 × 10^−13^
AST (IU/L) ^e^	20 (17–24)	19 (17–23)	23 (19–29)	24 (20–30)	26 (20–35)	<1.0 × 10^−13^	23 (20–28)	23 (19–27)	25 (20–31)	26 (22–33)	27 (22–36)	<1.0 × 10^−13^
CCI Score	0.77 ± 1.11	0.73 ± 1.07	0.99 ± 1.3	0.98 ± 1.24	1.84 ± 1.64	<1.0 × 10^−13^	1.42 ± 1.57	1.29 ± 1.49	1.7 ± 1.71	1.3 ± 1.44	2.87 ± 2.03	<1.0 × 10^−13^

Abbreviations: SLD, steatotic liver disease; MASLD, Metabolic dysfunction–associated steatotic liver disease; MetALD, metabolic alcohol-associated liver disease; ALD, alcohol-related liver disease; DM, diabetes mellitus; HBP, hypertension; OC, oral contraceptive; BMI, body mass index; SBP, systolic blood pressure; DBP, diastolic blood pressure; HDL-C, high-density lipoprotein cholesterol; LDL-C, low-density lipoprotein cholesterol; eGFR, estimated glomerular filtration rate; FLI, Fatty Liver Index; GGT, gamma-glutamyl transferase; ALT, alanine aminotransferase; AST, aspartate aminotransferase; CCI, Charlson Comorbidity Index. Values are expressed as mean ± SD or number (percentage) unless otherwise stated. ^a^. Defined as lowest income quartile and receiving public medical aid. ^b^. Alcohol consumption: non-drinker, moderate (<20 g/day), heavy (≥20 g/day). ^c^. Regular physical activity: moderate exercise ≥ 5 days/week or vigorous exercise ≥ 3 days/week. ^d^. Calculated with Modification of Diet in Renal Disease (MDRD) equation; eGFR = 186.3 × (Serum creatinine) − 1.154 × (Age) − 0.203 × 0.742. ^e^. Expressed as median (interquartile range, IQR).

**Table 2:** Reproductive and hormonal characteristics by SLD subtype in postmenopausal women.

Characteristics	Total N = 870,624	No SLD N = 753,210	MASLD N = 110,782	MetALD N = 4616	ALD N = 2016	*p*-Value
Age at menopause (years)	49.97 ± 3.97	49.94 ± 3.88	50.05 ± 4.17	49.92 ± 4.19	49.97 ± 4.21	<0.0001
Age at menopause						<0.0001
<40 40–44 45–49 50–54 ≥55	21,362 (1.75) 70,629 (5.8) 332,852 (27.34) 665,815 (54.68) 127,016 (10.43)	13,915 (1.65) 47,762 (5.65) 235,587 (27.88) 466,831 (55.25) 80,879 (9.57)	7268 (2) 22,288 (6.12) 94,886 (26.06) 194,518 (53.42) 45,137 (12.4)	92 (2.13) 284 (6.58) 1241 (28.77) 2231 (51.73) 465 (10.78)	87 (2.03) 295 (6.88) 1138 (26.53) 2235 (52.1) 535 (12.47)	
Total reproductive span						<0.0001
<30 30–34 35–39 ≥40	170,579 (14.01) 510,225 (41.9) 461,974 (37.94) 74,896 (6.15)	115,443 (13.66) 357,581 (42.32) 323,787 (38.32) 48,163 (5.7)	53,855 (14.79) 149,080 (40.95) 135,026 (37.09) 26,136 (7.18)	639 (14.82) 1773 (41.11) 1602 (37.14) 299 (6.93)	642 (14.97) 1791 (41.75) 1559 (36.34) 298 (6.95)	
HRT duration						<0.0001
Non <2 years 2–5 years ≥5 years	1,024,053 (84.1) 112,739 (9.26) 45,989 (3.78) 34,893 (2.87)	697,052 (82.49) 84,357 (9.98) 36,136 (4.28) 27,429 (3.25)	319,838 (87.84) 27,528 (7.56) 9519 (2.61) 7212 (1.98)	3527 (81.78) 467 (10.83) 183 (4.24) 136 (3.15)	3636 (84.76) 387 (9.02) 151 (3.52) 116 (2.7)	

Data are given as mean ± SD or number (percentage). *p* value was obtained by *t* test for continuous variables and χ2 test for categorical variables.

**Table 3:** Incidence and risk of gynecologic cancer in different SLD subtypes.

(A) In premenopause								
**Cancer** **Type**	**SLD** **Group**	**Participants,** **No.**	**Events** **No.**	**Duration,** **PY**	**IR ^a^,** **1000 PY**	**HR (95% CI)**		
						**Model 1 ^b^**	**Model 2 ^b^**	**Model 3 ^b^**
Cervical Cancer	No SLD	753,210	2240	9,237,032.48	0.24	1 (ref.)	1 (ref.)	1 (ref.)
	MASLD	110,782	376	1,355,047.71	0.28	1.14 (1.03, 1.28)	1.14 (1.03, 1.28)	1.13 (1.01, 1.26)
	MetALD	4616	28	56,187.35	0.50	2.06 (1.42, 2.99)	2.06 (1.42, 2.98)	1.87 (1.29, 2.73)
	ALD	2016	8	24,369.68	0.33	1.35 (0.68, 2.71)	1.35 (0.68, 2.71)	1.24 (0.62, 2.49)
	*p*-value					0.0002 ^c^	0.0002 ^c^	0.0019 ^c^
Endometrial Cancer	No SLD	753,210	2474	9,238,231.51	0.27	1 (ref.)	1 (ref.)	1 (ref.)
	MASLD	110,782	605	1,354,056.38	0.45	1.67 (1.53, 1.82)	1.61 (1.47, 1.76)	1.63 (1.50, 1.79)
	MetALD	4616	15	56,294.31	0.27	0.99 (0.60, 1.65)	0.98 (0.59, 1.63)	1.12 (0.67, 1.87)
	ALD	2016	10	24,389.65	0.41	1.53 (0.82, 2.86)	1.49 (0.80, 2.77)	1.67 (0.90, 3.11)
	*p*-value					<0.0001	<0.0001	<0.0001
Ovarian Cancer	No SLD	753,210	3704	9,234,548.06	0.40	1 (ref.)	1 (ref.)	1 (ref.)
	MASLD	110,782	663	1,354,322.13	0.49	1.22 (1.12, 1.33)	1.22 (1.12, 1.32)	1.22 (1.12, 1.33)
	MetALD	4616	26	56,245.90	0.46	1.15 (0.78, 1.70)	1.15 (0.78, 1.69)	1.17 (0.79, 1.72)
	ALD	2016	11	24,356.21	0.45	1.12 (0.62, 2.03)	1.12 (0.62, 2.03)	1.13 (0.62, 2.04)
	*p*-value					<0.0001	<0.0001	<0.0001
(B) In postmenopause								
**Cancer** **type**	**SLD** **group**	**Participants,** **No.**	**Events** **No.**	**Duration, PY**	**IR ^a^,** **1000 PY**		**HR (95% CI)**	
						**Model 1 ^b^**	**Model 2 ^b^**	**Model 3 ^b^**
Cervical Cancer	No SLD	844,974	2521	10,074,004.15	0.25	1 (ref.)	1 (ref.)	1 (ref.)
	MASLD	364,097	1254	4,303,481.77	0.29	1.16 (1.09, 1.25)	1.14 (1.07, 1.22)	1.12 (1.05, 1.20)
	MetALD	4313	19	51,860.00	0.37	1.47 (0.93, 2.30)	1.51 (0.96, 2.37)	1.38 (0.88, 2.17)
	ALD	4290	14	49,986.73	0.28	1.12 (0.66, 1.89)	1.12 (0.66, 1.89)	1.06 (0.63, 1.79)
	*p*-value					<0.0001 ^c^	0.0006 ^c^	0.0068 ^c^
Endometrial Cancer	No SLD	844,974	1803	10,077,950.13	0.18	1 (ref.)	1 (ref.)	1 (ref.)
	MASLD	364,097	1007	4,304,761.84	0.23	1.31 (1.21, 1.41)	1.40 (1.29, 1.52)	1.42 (1.32, 1.54)
	MetALD	4313	10	51,919.29	0.19	1.08 (0.58, 2.00)	0.98 (0.53, 1.83)	1.04 (0.56, 1.93)
	ALD	4290	7	50,002.92	0.14	0.78 (0.37, 1.65)	0.79 (0.38, 1.65)	0.82 (0.39, 1.71)
	*p*-value					<0.0001	<0.0001	<0.0001
Ovarian Cancer	No SLD	844,974	3909	10,072,991.40	0.39	1 (ref.)	1 (ref.)	1 (ref.)
	MASLD	364,097	1895	4,303,366.77	0.44	1.14 (1.08, 1.20)	1.13 (1.07, 1.19)	1.14 (1.08, 1.20)
	MetALD	4313	20	51,919.04	0.39	0.99 (0.64, 1.54)	1.01 (0.65, 1.56)	0.98 (0.63, 1.52)
	ALD	4290	16	50,022.85	0.32	0.83 (0.51, 1.35)	0.83 (0.51, 1.35)	0.82 (0.50, 1.34)
	*p*-value					<0.0001	0.0003	<0.0001

^a^. Incidence per 1000 person years. ^b^. Model 1: Non-adjusted. Model 2: Adjusted for age. Model 3 (in pre-menopause): Adjusted for age, income level, parity, feed, oral contraceptives, smoking status, and regular exercise, age at menarch. Model 3 (in post-menopause): Adjusted for age, income level, parity, feed, oral contraceptives, smoking status, and regular exercise, age at menarch, age at menopause, HRT. ^c^. *p* for trend.

## Data Availability

We obtained health insurance claims data from the Health Insurance Review and Assessment Service (HIRA) of South Korea for use in our research, and these data are accessible to anyone through approval from the institution. The data provided by HIRA were anonymized and stripped of personally identifiable information before being made available. The datasets used and/or analyzed during the current study are available from the corresponding author on reasonable request.

## Supplementary Materials

The following supporting information can be downloaded at: https://www.mdpi.com/article/10.3390/cancers18060894/s1, Table S1: Wald test *p*-value represents the significance of each covariate in the multivariate model (Model 3); Table S2: Association between MASLD and the risk of gynecologic cancers in the total study population; Table S3: Risk of gynecologic cancers associated with MASLD stratified by menopausal status and reproductive/lifestyle factors; Table S4: Sensitivity analysis restricting the follow-up period to age 55 years; Table S5: Sensitivity analysis; Table S6: Forest plots of subgroup analyses by menopausal status and cancer type.

## Abbreviations

The following abbreviations are used in this manuscript:

SLD	Steatotic liver disease
MASLD	Metabolic dysfunction-associated steatotic liver disease
MetALD	Metabolic alcohol-associated liver disease
ALD	Alcohol-related liver disease
aHR	Adjusted hazard ratios
CI	Confidence interval
PY	Person-years
IR	Incidence rate
HRT	Hormone replacement therapy
OC	Oral contraceptive
