# Supplementary material for: Metabolic Dysfunction-Associated Steatotic Liver Disease (MASLD) and Risk of Gynecologic Cancer: A Nationwide Cohort Study
Source: Cancers (Basel). 2026 Mar 10;18(6):894. doi: 10.3390/cancers18060894 (PMC13025304; doi:10.3390/cancers18060894)

Supplementary Table S1. Wald test p-value represents the significance of each covariate in the multivariate model (Model 3).

Variable	In Pre-menopause			In Post-menopause		
	Cervical cancer	Endometrial cancer	Ovarian cancer	Cervical cancer	Endometrial cancer	Ovarian cancer
SLD Group	0.0019	<.0001	<.0001	0.0068	<.0001	<.0001
Age	0.2628	<.0001	0.0143	0.0054	<.0001	<.0001
Income	0.0717	0.4239	0.2098	0.005	0.0291	0.9478
Smoking	0.0013	0.0001	0.6542	0.0004	0.0046	0.1362
Regular exercise	0.7238	0.0683	0.2783	0.617	0.0097	0.5147
Parity	0.8512	<.0001	<.0001	0.5193	<.0001	0.0002
Feed	0.1637	0.0643	0.0097	0.0036	0.0168	0.158
OC	0.6454	0.0025	0.2805	0.4036	0.9757	0.5341
Age at Menarche	0.0019	0.0006	0.0008	0.0753	<.0001	0.067
Age at Menopause	-	-	-	0.394	<.0001	<.0001
HRT	-	-	-	0.0256	0.0002	0.0027

Supplementary Table S2. Association between MASLD and the risk of gynecologic cancers in the total study population

Cancer Type	SLD Group	N	Event	Duration, PY	IR, 1000 PY	Model 1	Model 2	Model 3
Cervical cancer	No SLD	1598184	4761	19311036.63	0.25	1 (ref.)	1 (ref.)	1 (ref.)
	MASLD	474879	1630	5658529.48	0.29	1.168 (1.104, 1.236)	1.138 (1.075, 1.206)	**1.124 (1.061, 1.191)**
	MetALD	8929	47	108047.35	0.43	1.743 (1.308, 2.323)	1.759 (1.32, 2.345)	**1.606 (1.202, 2.145)**
	ALD	6306	22	74356.41	0.30	1.243 (0.825, 1.872)	1.228 (0.815, 1.849)	1.155 (0.766, 1.742)
	p-value					<.0001	<.0001	<.0001
Endometrial cancer	No SLD	1598184	4277	19316181.64	0.22	1 (ref.)	1 (ref.)	1 (ref.)
	MASLD	474879	1612	5658818.22	0.28	1.289 (1.217, 1.364)	1.48 (1.395, 1.57)	**1.509 (1.422, 1.601)**
	MetALD	8929	25	108213.60	0.23	1.071 (0.729, 1.575)	1.034 (0.703, 1.52)	1.144 (0.777, 1.684)
	ALD	6306	17	74392.57	0.23	1.025 (0.637, 1.651)	1.099 (0.682, 1.769)	1.182 (0.734, 1.905)
	p-value					<.0001	<.0001	<.0001
Ovarian cancer	No SLD	1598184	7613	19307539.46	0.39	1 (ref.)	1 (ref.)	1 (ref.)
	MASLD	474879	2558	5657688.90	0.45	1.149 (1.099, 1.202)	1.15 (1.098, 1.204)	**1.158 (1.106, 1.213)**
	MetALD	8929	46	108164.94	0.43	1.134 (0.857, 1.502)	1.134 (0.857, 1.502)	1.123 (0.847, 1.489)
	ALD	6306	27	74379.06	0.36	0.947 (0.654, 1.373)	0.948 (0.654, 1.373)	0.942 (0.65, 1.366)
	p-value					<.0001	<.0001	<.0001

Model 1: Non-adjusted, Model 2: Age, Model 3: Age, Income, Smoking, Regular exercise, Parity, Feed, OC, Age at Menarche

Supplementary Table S3. Risk of gynecologic cancers associated with MASLD stratified by menopausal status and reproductive/lifestyle factors

1) In Premenopause

Subgroup	Group	N	**Cervical**	Duration	IR per 1,000	Model 3	p value	**Endometrial**	Duration	IR per 1,000	Model 3	p value	**Ovarian**	Duration	IR per 1,000	Model 3	p value
Smoking, Non-Ex	No SLD	730463	2161	8959468.84	0.24	1 (ref.)	0.547	2421	8960499.97	0.27	1 (ref.)	0.652	3594	8957020.43	0.40	1 (ref.)	0.528
	MASLD	106315	355	1300715.51	0.27	1.119 (0.999, 1.253)		593	1299634.02	0.46	1.65 (1.506, 1.807)		633	1299941.93	0.49	1.212 (1.113, 1.32)	
	MetALD	3318	21	40514.18	0.52	2.028 (1.318, 3.119)		11	40598.64	0.27	1.03 (0.569, 1.863)		18	40548.79	0.44	1.114 (0.701, 1.771)	
	ALD	1466	7	17753.05	0.39	1.555 (0.74, 3.267)		8	17774.57	0.45	1.663 (0.83, 3.329)		10	17739.51	0.56	1.406 (0.756, 2.616)	
Smoking, Current	No SLD	22747	79	277563.64	0.28	1 (ref.)		53	277731.54	0.19	1 (ref.)		110	277527.63	0.40	1 (ref.)	
	MASLD	4467	21	54332.21	0.39	1.344 (0.831, 2.175)		12	54422.36	0.22	1.151 (0.615, 2.154)		30	54380.20	0.55	1.409 (0.941, 2.11)	
	MetALD	1298	7	15673.16	0.45	1.532 (0.707, 3.32)		4	15695.67	0.25	1.397 (0.506, 3.861)		8	15697.11	0.51	1.33 (0.649, 2.726)	
	ALD	550	1	6616.64	0.15	0.518 (0.072, 3.723)		2	6615.08	0.30	1.612 (0.393, 6.614)		1	6616.70	0.15	0.387 (0.054, 2.764)	
Regular exercise, No	No SLD	621160	1843	7615189.07	0.24	1 (ref.)	0.573	1989	7616473.85	0.26	1 (ref.)	0.141	3071	7613073.23	0.40	1 (ref.)	0.83
	MASLD	93990	326	1149349.85	0.28	1.158 (1.028, 1.304)		524	1148439.31	0.46	1.708 (1.55, 1.883)		572	1148664.41	0.50	1.235 (1.129, 1.351)	
	MetALD	3808	25	46298.16	0.54	2.033 (1.364, 3.03)		11	46403.03	0.24	1.025 (0.565, 1.857)		22	46363.24	0.47	1.191 (0.781, 1.816)	
	ALD	1682	8	20316.46	0.39	1.492 (0.743, 2.996)		9	20337.03	0.44	1.841 (0.955, 3.549)		10	20308.08	0.49	1.22 (0.655, 2.273)	
Regular exercise, Yes	No SLD	132050	397	1621843.41	0.24	1 (ref.)		485	1621757.66	0.30	1 (ref.)		633	1621474.84	0.39	1 (ref.)	
	MASLD	16792	50	205697.86	0.24	0.977 (0.728, 1.311)		81	205617.08	0.39	1.296 (1.024, 1.64)		91	205657.72	0.44	1.136 (0.912, 1.416)	
	MetALD	808	3	9889.19	0.30	1.127 (0.362, 3.515)		4	9891.28	0.40	1.504 (0.562, 4.026)		4	9882.65	0.40	1.046 (0.391, 2.799)	
	ALD	334	0	4053.22	0.00	-		1	4052.61	0.25	0.914 (0.128, 6.501)		1	4048.13	0.25	0.643 (0.09, 4.573)	
Age at menarche ≤ 12	No SLD	33077	89	404826.28	0.22	1 (ref.)	0.724	121	404720.15	0.30	1 (ref.)	0.882	206	404428.37	0.51	1 (ref.)	0.998
	MASLD	5948	13	72659.95	0.18	0.81 (0.453, 1.45)		38	72516.38	0.52	1.675 (1.163, 2.413)		45	72537.85	0.62	1.204 (0.872, 1.663)	
	MetALD	185	1	2241.33	0.45	1.818 (0.253, 13.069)		0	2252.97	0.00	-		0	2252.97	0.00	-	
	ALD	86	0	991.88	0.00	-		1	988.18	1.01	3.664 (0.512, 26.234)		0	991.88	0.00	-	
Age at menarche > 12	No SLD	720133	2151	8832206.20	0.24	1 (ref.)		2353	8833511.35	0.27	1 (ref.)		3498	8830119.69	0.40	1 (ref.)	
	MASLD	104834	363	1282387.76	0.28	1.145 (1.023, 1.281)		567	1281540.00	0.44	1.634 (1.489, 1.793)		618	1281784.29	0.48	1.221 (1.12, 1.331)	
	MetALD	4431	27	53946.02	0.50	1.874 (1.277, 2.751)		15	54041.34	0.28	1.17 (0.703, 1.949)		26	53992.92	0.48	1.229 (0.834, 1.812)	
	ALD	1930	8	23377.81	0.34	1.29 (0.643, 2.589)		9	23401.47	0.38	1.575 (0.817, 3.035)		11	23364.33	0.47	1.189 (0.657, 2.153)	
OC, <1 years	No SLD	729000	2161	8940174.96	0.24	1 (ref.)	0.790	2420	8941175.16	0.27	1 (ref.)	0.958	3594	8937673.53	0.40	1 (ref.)	0.891
	MASLD	106021	355	1296762.25	0.27	1.119 (0.999, 1.253)		589	1295725.57	0.45	1.64 (1.497, 1.796)		641	1295985.56	0.49	1.227 (1.127, 1.336)	
	MetALD	4143	26	50470.70	0.52	1.954 (1.322, 2.888)		14	50565.68	0.28	1.122 (0.662, 1.901)		24	50519.64	0.48	1.187 (0.793, 1.778)	
	ALD	1819	8	21982.84	0.36	1.393 (0.694, 2.794)		9	22006.51	0.41	1.606 (0.834, 3.095)		11	21969.36	0.50	1.242 (0.686, 2.248)	

OC, ≥1 year	No SLD	24210	79	296857.52	0.27	1 (ref.)		54	297056.34	0.18	1 (ref.)		110	296874.53	0.37	1 (ref.)	
	MASLD	4761	21	58285.47	0.36	1.348 (0.833, 2.182)		16	58330.81	0.27	1.503 (0.86, 2.626)		22	58336.57	0.38	1.034 (0.654, 1.635)	
	MetALD	473	2	5716.65	0.35	1.215 (0.298, 4.951)		1	5728.63	0.17	1.082 (0.149, 7.824)		2	5726.26	0.35	0.933 (0.23, 3.783)	
	ALD	197	0	2386.84	0.00	-		1	2383.14	0.42	2.583 (0.357, 18.694)		0	2386.84	0.00	-	
Parity, No	No SLD	27851	78	340198.39	0.23	1 (ref.)	0.513	133	340017.74	0.39	1 (ref.)	<.0001	204	339735.96	0.60	1 (ref.)	0.679
	MASLD	3446	11	41843.45	0.26	1.144 (0.608, 2.151)		57	41602.01	1.37	3.45 (2.529, 4.706)		38	41741.41	0.91	1.504 (1.063, 2.126)	
	MetALD	218	3	2603.74	1.15	4.472 (1.408, 14.209)		1	2616.22	0.38	1.199 (0.167, 8.586)		0	2617.63	0.00	-	
	ALD	118	0	1419.53	0.00	-		0	1419.53	0.00	-		0	1419.53	0.00	-	
Parity, Yes	No SLD	725359	2162	8896834.09	0.24	1 (ref.)		2341	8898213.77	0.26	1 (ref.)		3500	8894812.10	0.39	1 (ref.)	
	MASLD	107336	365	1313204.26	0.28	1.129 (1.009, 1.262)		548	1312454.37	0.42	1.548 (1.409, 1.7)		625	1312580.72	0.48	1.206 (1.106, 1.314)	
	MetALD	4398	25	53583.61	0.47	1.75 (1.175, 2.607)		14	53678.09	0.26	1.116 (0.659, 1.891)		26	53628.27	0.48	1.251 (0.849, 1.844)	
	ALD	1898	8	22950.16	0.35	1.32 (0.658, 2.648)		10	22970.12	0.44	1.808 (0.97, 3.37)		11	22936.68	0.48	1.23 (0.68, 2.227)	
Breast Feeding, No	No SLD	135475	389	1658877.80	0.23	1 (ref.)	0.866	489	1659003.95	0.29	1 (ref.)	<.0001	777	1657918.81	0.47	1 (ref.)	0.040
	MASLD	18713	67	228298.90	0.29	1.247 (0.962, 1.617)		167	227872.34	0.73	2.489 (2.087, 2.968)		151	228039.34	0.66	1.414 (1.188, 1.684)	
	MetALD	934	6	11327.83	0.53	2.033 (0.905, 4.565)		4	11352.94	0.35	1.4 (0.522, 3.751)		2	11354.25	0.18	0.38 (0.095, 1.524)	
	ALD	454	0	5473.81	0.00	-		1	5470.11	0.18	0.721 (0.101, 5.137)		1	5462.79	0.18	0.39 (0.055, 2.776)	
Breast Feeding, Yes	No SLD	617735	1851	7578154.68	0.24	1 (ref.)		1985	7579227.55	0.26	1 (ref.)		2927	7576629.26	0.39	1 (ref.)	
	MASLD	92069	309	1126748.81	0.27	1.106 (0.98, 1.249)		438	1126184.05	0.39	1.441 (1.298, 1.6)		512	1126282.79	0.45	1.172 (1.066, 1.288)	
	MetALD	3682	22	44859.52	0.49	1.831 (1.198, 2.798)		11	44941.37	0.24	1.045 (0.577, 1.893)		24	44891.65	0.53	1.408 (0.94, 2.108)	
	ALD	1562	8	18895.87	0.42	1.596 (0.796, 3.202)		9	18919.54	0.48	1.962 (1.018, 3.78)		10	18893.42	0.53	1.39 (0.746, 2.589)	

2) In Postmenopause

Subgroup	Group	N	**Cervical**	Duration	IR per 1,000	Model 3	p value	**Corpus**	Duration	IR per 1,000	Model 3	p value	**Ovarian**	Duration	IR per 1,000	Model 3	p value
Smoking, Non-Ex	No SLD	824188	2449	9836629.80	0.25	1 (ref.)	0.309	1773	9840337.40	0.18	1 (ref.)	0.933	3800	9835631.29	0.39	1 (ref.)	0.651
	MASLD	353950	1204	4187831.55	0.29	1.112 (1.038, 1.193)		985	4188923.21	0.24	1.42 (1.311, 1.537)		1840	4187585.25	0.44	1.139 (1.077, 1.205)	
	MetALD	3391	14	40832.55	0.34	1.369 (0.809, 2.317)		10	40869.41	0.24	1.243 (0.667, 2.315)		13	40887.91	0.32	0.832 (0.483, 1.435)	
	ALD	3797	10	44422.09	0.23	0.878 (0.472, 1.635)		6	44423.60	0.14	0.77 (0.345, 1.716)		16	44437.88	0.36	0.937 (0.573, 1.531)	
Smoking, Current	No SLD	20786	72	237374.35	0.30	1 (ref.)		30	237612.73	0.13	1 (ref.)		109	237360.11	0.46	1 (ref.)	
	MASLD	10147	50	115650.23	0.43	1.375 (0.958, 1.972)		22	115838.63	0.19	1.628 (0.939, 2.823)		55	115781.52	0.48	1.046 (0.756, 1.447)	
	MetALD	922	5	11027.45	0.45	1.537 (0.621, 3.806)		0	11049.88	0.00	-		7	11031.13	0.63	1.399 (0.651, 3.004)	

	ALD	493	4	5564.63	0.72	2.382 (0.87, 6.519)		1	5579.32	0.18	1.292 (0.176, 9.474)		0	5584.97	0.00	-
Regular exercise, No	No SLD	683193	2056	8115541.89	0.25	1 (ref.)	0.683	1397	8119118.59	0.17	1 (ref.)	0.929	3139	8114906.49	0.39	1 (ref.)	0.921
	MASLD	305154	1045	3596866.58	0.29	1.106 (1.026, 1.193)		817	3598077.85	0.23	1.425 (1.306, 1.555)		1570	3596950.49	0.44	1.128 (1.061, 1.2)	
	MetALD	3479	17	41795.12	0.41	1.51 (0.935, 2.44)		8	41849.52	0.19	1.068 (0.532, 2.143)		16	41855.78	0.38	0.969 (0.592, 1.586)	
	ALD	3596	12	41776.89	0.29	1.075 (0.609, 1.896)		5	41791.18	0.12	0.718 (0.298, 1.728)		14	41806.72	0.33	0.857 (0.507, 1.449)	
Regular exercise, Yes	No SLD	161781	465	1958462.25	0.24	1 (ref.)		406	1958831.54	0.21	1 (ref.)		770	1958084.91	0.39	1 (ref.)	
	MASLD	58943	209	706615.19	0.30	1.193 (1.013, 1.405)		190	706683.99	0.27	1.417 (1.192, 1.684)		325	706416.28	0.46	1.173 (1.03, 1.336)	
	MetALD	834	2	10064.88	0.20	0.794 (0.198, 3.186)		2	10069.77	0.20	0.927 (0.231, 3.723)		4	10063.26	0.40	1.001 (0.374, 2.674)	
	ALD	694	2	8209.84	0.24	0.981 (0.245, 3.935)		2	8211.75	0.24	1.231 (0.307, 4.941)		2	8216.13	0.24	0.617 (0.154, 2.471)	
Age at menarche ≤ 12	No SLD	8459	33	102597.28	0.32	1 (ref.)	0.985	24	102637.31	0.23	1 (ref.)	0.261	51	102544.12	0.50	1 (ref.)	0.975
	MASLD	3156	13	38014.88	0.34	0.998 (0.525, 1.897)		9	38042.72	0.24	1.114 (0.518, 2.398)		19	38060.67	0.50	1.013 (0.598, 1.715)	
	MetALD	58	0	716.23	0.00	-		1	711.22	1.41	6.663 (0.909, 48.831)		0	716.23	0.00	-	
	ALD	60	0	700.83	0.00	-		0	700.83	0.00	-		0	700.83	0.00	-	
Age at menarche > 12	No SLD	836515	2488	9971406.87	0.25	1 (ref.)		1779	9975312.81	0.18	1 (ref.)		3858	9970447.28	0.39	1 (ref.)	
	MASLD	360941	1241	4265466.89	0.29	1.122 (1.047, 1.202)		998	4266719.12	0.23	1.427 (1.319, 1.544)		1876	4265306.10	0.44	1.138 (1.076, 1.203)	
	MetALD	4255	19	51143.77	0.37	1.405 (0.893, 2.211)		9	51208.07	0.18	0.948 (0.491, 1.827)		20	51202.81	0.39	0.992 (0.638, 1.542)	
	ALD	4230	14	49285.90	0.28	1.081 (0.639, 1.829)		7	49302.10	0.14	0.828 (0.394, 1.74)		16	49322.02	0.32	0.833 (0.51, 1.361)	
OC, <1 years	No SLD	796012	2353	9483031.68	0.25	1 (ref.)	0.314	1693	9486621.03	0.18	1 (ref.)	0.899	3697	9481916.45	0.39	1 (ref.)	0.099
	MASLD	338956	1170	4003428.44	0.29	1.135 (1.057, 1.218)		939	4004591.69	0.23	1.431 (1.319, 1.551)		1765	4003320.25	0.44	1.131 (1.068, 1.198)	
	MetALD	3871	15	46507.76	0.32	1.23 (0.739, 2.047)		10	46549.72	0.21	1.164 (0.624, 2.172)		18	46557.87	0.39	0.973 (0.612, 1.549)	
	ALD	3948	14	45954.97	0.30	1.167 (0.69, 1.974)		6	45980.82	0.13	0.762 (0.342, 1.7)		12	46004.72	0.26	0.663 (0.376, 1.169)	
OC, ≥1 year	No SLD	48962	168	590972.47	0.28	1 (ref.)		110	591329.10	0.19	1 (ref.)		212	591074.94	0.36	1 (ref.)	
	MASLD	25141	84	300053.33	0.28	0.946 (0.727, 1.229)		68	300170.15	0.23	1.324 (0.978, 1.793)		130	300046.52	0.43	1.22 (0.98, 1.518)	
	MetALD	442	4	5352.23	0.75	2.47 (0.915, 6.667)		0	5369.57	0.00	-		2	5361.16	0.37	1.01 (0.251, 4.068)	
	ALD	342	0	4031.75	0.00	-		1	4022.10	0.25	1.379 (0.192, 9.877)		4	4018.13	1.00	2.757 (1.029, 7.384)	
Parity, No	No SLD	15267	38	183670.80	0.21	1 (ref.)	0.696	59	183559.20	0.32	1 (ref.)	0.969	121	183401.45	0.66	1 (ref.)	0.584
	MASLD	4686	13	55535.50	0.23	1.089 (0.58, 2.044)		26	55454.17	0.47	1.593 (1.004, 2.528)		32	55481.17	0.58	0.866 (0.586, 1.278)	
	MetALD	130	0	1561.41	0.00	-		0	1561.41	0.00	-		0	1561.41	0.00	-	
	ALD	110	1	1244.49	0.80	3.466 (0.475, 25.268)		0	1248.19	0.00	-		0	1248.19	0.00	-	
Parity, Yes	No SLD	829707	2483	9890333.35	0.25	1 (ref.)		1744	9894390.92	0.18	1 (ref.)		3788	9889589.94	0.38	1 (ref.)	
	MASLD	359411	1241	4247946.28	0.29	1.121 (1.046, 1.201)		981	4249307.67	0.23	1.419 (1.311, 1.537)		1863	4247885.60	0.44	1.143 (1.081, 1.209)	

	MetALD	4183	19	50298.59	0.38	1.417 (0.901, 2.231)		10	50357.88	0.20	1.085 (0.582, 2.024)		20	50357.63	0.40	1.026 (0.66, 1.594)	
	ALD	4180	13	48742.24	0.27	1.007 (0.584, 1.737)		7	48754.74	0.14	0.852 (0.405, 1.79)		16	48774.66	0.33	0.853 (0.522, 1.395)	
Breast Feeding, No	No SLD	58301	141	706107.49	0.20	1 (ref.)	0.989	183	705839.70	0.26	1 (ref.)	0.957	331	705453.24	0.47	1 (ref.)	0.974
	MASLD	18247	53	218765.90	0.24	1.169 (0.852, 1.604)		80	218622.63	0.37	1.526 (1.173, 1.985)		120	218555.13	0.55	1.161 (0.942, 1.43)	
	MetALD	444	0	5372.64	0.00	-		0	5372.64	0.00	-		2	5369.57	0.37	0.765 (0.19, 3.075)	
	ALD	330	1	3817.49	0.26	1.187 (0.166, 8.493)		0	3821.19	0.00	-		0	3821.19	0.00	-	
Breast Feeding, Yes	No SLD	786673	2380	9367896.66	0.25	1 (ref.)		1620	9372110.43	0.17	1 (ref.)		3578	9367538.15	0.38	1 (ref.)	
	MASLD	345850	1201	4084715.88	0.29	1.118 (1.043, 1.2)		927	4086139.21	0.23	1.415 (1.303, 1.535)		1775	4084811.64	0.43	1.135 (1.071, 1.202)	
	MetALD	3869	19	46487.35	0.41	1.514 (0.962, 2.382)		10	46546.64	0.21	1.192 (0.639, 2.223)		18	46549.47	0.39	1.006 (0.632, 1.6)	
	ALD	3960	13	46169.23	0.28	1.052 (0.61, 1.815)		7	46181.73	0.15	0.915 (0.435, 1.923)		16	46201.66	0.35	0.905 (0.553, 1.478)	
Age at menopause < 40	No SLD	13915	51	161811.81	0.32	1 (ref.)	0.212	15	161986.40	0.09	1 (ref.)	0.996	53	161919.40	0.33	1 (ref.)	0.942
	MASLD	7268	17	84032.87	0.20	0.623 (0.36, 1.079)		9	84057.04	0.11	1.305 (0.571, 2.984)		27	83992.56	0.32	0.988 (0.622, 1.571)	
	MetALD	92	0	1089.87	0.00	-		0	1089.87	0.00	-		0	1089.87	0.00	-	
	ALD	87	0	995.39	0.00	-		0	995.39	0.00	-		0	995.39	0.00	-	
Age at menopause ≥ 40	No SLD	831059	2470	9912192.33	0.25	1 (ref.)		1788	9915963.72	0.18	1 (ref.)		3856	9911071.99	0.39	1 (ref.)	
	MASLD	356829	1237	4219448.91	0.29	1.132 (1.057, 1.213)		998	4220704.80	0.24	1.425 (1.317, 1.541)		1868	4219374.21	0.44	1.139 (1.077, 1.204)	
	MetALD	4221	19	50770.13	0.37	1.413 (0.898, 2.225)		10	50829.42	0.20	1.05 (0.563, 1.958)		20	50829.17	0.39	0.993 (0.639, 1.543)	
	ALD	4203	14	48991.34	0.29	1.086 (0.642, 1.838)		7	49007.53	0.14	0.824 (0.392, 1.732)		16	49027.46	0.33	0.831 (0.509, 1.359)	
HRT < 2 years	No SLD	781409	2373	9297653.88	0.26	1 (ref.)	0.288	1601	9301801.07	0.17	1 (ref.)	0.011	3548	9297227.18	0.38	1 (ref.)	0.188
	MASLD	347366	1194	4100807.74	0.29	1.105 (1.03, 1.185)		966	4102007.72	0.24	1.477 (1.362, 1.601)		1810	4100725.72	0.44	1.152 (1.088, 1.22)	
	MetALD	3994	19	47970.95	0.40	1.465 (0.93, 2.305)		9	48032.05	0.19	1.043 (0.541, 2.012)		19	48031.60	0.40	1.018 (0.648, 1.6)	
	ALD	4023	14	46776.05	0.30	1.115 (0.659, 1.887)		6	46801.90	0.13	0.777 (0.348, 1.733)		14	46822.14	0.30	0.776 (0.459, 1.313)	
HRT ≥ 2 years	No SLD	63565	148	776350.27	0.19	1 (ref.)		202	776149.06	0.26	1 (ref.)		361	775764.21	0.47	1 (ref.)	
	MASLD	16731	60	202674.03	0.30	1.497 (1.109, 2.021)		41	202754.12	0.20	0.826 (0.59, 1.156)		85	202641.05	0.42	0.903 (0.713, 1.143)	
	MetALD	319	0	3889.04	0.00	-		1	3887.23	0.26	0.983 (0.138, 7.015)		1	3887.44	0.26	0.544 (0.076, 3.872)	
	ALD	267	0	3210.68	0.00	-		1	3201.02	0.31	1.169 (0.164, 8.343)		2	3200.71	0.62	1.313 (0.327, 5.27)	

Supplementary Table S4. Sensitivity analysis restricting the follow-up period to age 55 years

	**In Premenopause**						**In Premenopause ((follow up until age 55))**					
	Total	No SLD	MASLD	MetALD	ALD	p-value	Total	No SLD	MASLD	MetALD	ALD	p-value
n	870624	753210	110782	4616	2016		859548	745610	107398	4571	1969	
Age ≥ 65	2425 (0.28)	1560 (0.21)	856 (0.77)	3 (0.06)	6 (0.3)	<.0001						-
Income 25% + MA	215926 (24.8)	186436 (24.75)	27628 (24.94)	1269 (27.49)	593 (29.41)	<.0001	213225 (24.81)	184578 (24.76)	26813 (24.97)	1253 (27.41)	581 (29.51)	<.0001
Smoking						<.0001						<.0001
Non	827813 (95.08)	718821 (95.43)	104563 (94.39)	3050 (66.07)	1379 (68.4)		817075 (95.06)	711426 (95.42)	101294 (94.32)	3014 (65.94)	1341 (68.11)	
Ex	13749 (1.58)	11642 (1.55)	1752 (1.58)	268 (5.81)	87 (4.32)		13629 (1.59)	11568 (1.55)	1710 (1.59)	265 (5.8)	86 (4.37)	
Current	29062 (3.34)	22747 (3.02)	4467 (4.03)	1298 (28.12)	550 (27.28)		28844 (3.36)	22616 (3.03)	4394 (4.09)	1292 (28.27)	542 (27.53)	
Drinking						<.0001						<.0001
Non	623930 (71.66)	541978 (71.96)	81396 (73.47)	0 (0)	556 (27.58)		614323 (71.47)	535386 (71.81)	78410 (73.01)	0 (0)	527 (26.76)	
Mild	225103 (25.86)	195354 (25.94)	29386 (26.53)	0 (0)	363 (18.01)		223766 (26.03)	194419 (26.08)	28988 (26.99)	0 (0)	359 (18.23)	
Heavy	21591 (2.48)	15878 (2.11)	0 (0)	4616 (100)	1097 (54.41)		21459 (2.5)	15805 (2.12)	0 (0)	4571 (100)	1083 (55)	
Regular exercise	149984 (17.23)	132050 (17.53)	16792 (15.16)	808 (17.5)	334 (16.57)	<.0001	148079 (17.23)	130659 (17.52)	16297 (15.17)	800 (17.5)	323 (16.4)	<.0001
DM	30451 (3.5)	16629 (2.21)	13078 (11.81)	461 (9.99)	283 (14.04)	<.0001	29121 (3.39)	16007 (2.15)	12394 (11.54)	451 (9.87)	269 (13.66)	<.0001
HP	121266 (13.93)	82109 (10.9)	36574 (33.01)	1708 (37)	875 (43.4)	<.0001	116171 (13.52)	79167 (10.62)	34477 (32.1)	1684 (36.84)	843 (42.81)	<.0001
DYS	93373 (10.72)	64251 (8.53)	27495 (24.82)	1018 (22.05)	609 (30.21)	<.0001	89734 (10.44)	62136 (8.33)	26013 (24.22)	999 (21.86)	586 (29.76)	<.0001
Age at menarche, years	15.09 ± 1.68	15.07 ± 1.66	15.18 ± 1.8	15.42 ± 1.8	15.47 ± 1.78	<.0001	15.08 ± 1.67	15.06 ± 1.65	15.15 ± 1.78	15.41 ± 1.8	15.45 ± 1.77	<.0001
Age at menarche						<.0001						<.0001
≤12	39296 (4.51)	33077 (4.39)	5948 (5.37)	185 (4.01)	86 (4.27)		38539 (4.48)	32597 (4.37)	5673 (5.28)	185 (4.05)	84 (4.27)	
≤14	274932 (31.58)	240195 (31.89)	33066 (29.85)	1189 (25.76)	482 (23.91)		273617 (31.83)	239260 (32.09)	32697 (30.44)	1182 (25.86)	478 (24.28)	
≤16	398570 (45.78)	347412 (46.12)	48167 (43.48)	2091 (45.3)	900 (44.64)		394348 (45.88)	344506 (46.2)	46888 (43.66)	2073 (45.35)	881 (44.74)	
>16	157826 (18.13)	132526 (17.59)	23601 (21.3)	1151 (24.94)	548 (27.18)		153044 (17.81)	129247 (17.33)	22140 (20.61)	1131 (24.74)	526 (26.71)	
OC						<.0001						<.0001
Non	758214 (87.09)	658270 (87.4)	94928 (85.69)	3468 (75.13)	1548 (76.79)		748559 (87.09)	651643 (87.4)	91975 (85.64)	3431 (75.06)	1510 (76.69)	
<1 year	82769 (9.51)	70730 (9.39)	11093 (10.01)	675 (14.62)	271 (13.44)		81888 (9.53)	70123 (9.4)	10826 (10.08)	671 (14.68)	268 (13.61)	
≥1 year	29641 (3.4)	24210 (3.21)	4761 (4.3)	473 (10.25)	197 (9.77)		29101 (3.39)	23844 (3.2)	4597 (4.28)	469 (10.26)	191 (9.7)	
Parity						<.0001						<.0001
0	31633 (3.63)	27851 (3.7)	3446 (3.11)	218 (4.72)	118 (5.85)		31462 (3.66)	27719 (3.72)	3408 (3.17)	217 (4.75)	118 (5.99)	
1	115182 (13.23)	99797 (13.25)	14257 (12.87)	784 (16.98)	344 (17.06)		112618 (13.1)	98113 (13.16)	13393 (12.47)	778 (17.02)	334 (16.96)	

≥2	723809 (83.14)	625562 (83.05)	93079 (84.02)	3614 (78.29)	1554 (77.08)		715468 (83.24)	619778 (83.12)	90597 (84.36)	3576 (78.23)	1517 (77.04)	
Breast Feeding						<.0001						<.0001
No	155576 (17.87)	135475 (17.99)	18713 (16.89)	934 (20.23)	454 (22.52)		154885 (18.02)	134969 (18.1)	18532 (17.26)	932 (20.39)	452 (22.96)	
<6 months	214119 (24.59)	191909 (25.48)	21001 (18.96)	872 (18.89)	337 (16.72)		211215 (24.57)	189982 (25.48)	20042 (18.66)	867 (18.97)	324 (16.46)	
6-12 months	229559 (26.37)	200971 (26.68)	27147 (24.5)	1007 (21.82)	434 (21.53)		227406 (26.46)	199389 (26.74)	26589 (24.76)	1001 (21.9)	427 (21.69)	
≥12 months	271370 (31.17)	224855 (29.85)	43921 (39.65)	1803 (39.06)	791 (39.24)		266042 (30.95)	221270 (29.68)	42235 (39.33)	1771 (38.74)	766 (38.9)	
Age	45.08 ± 4.23	44.92 ± 4.1	46.16 ± 4.86	45.53 ± 3.98	46.04 ± 4.33	<.0001	44.88 ± 3.81	44.76 ± 3.77	45.69 ± 4	45.42 ± 3.8	45.73 ± 3.85	<.0001
Height	157.51 ± 5.16	157.6 ± 5.13	156.91 ± 5.34	157.28 ± 5.2	157.19 ± 5.29	<.0001	157.55 ± 5.14	157.63 ± 5.11	157 ± 5.31	157.32 ± 5.19	157.25 ± 5.28	<.0001
Weight	57.61 ± 8.08	55.94 ± 6.54	68.44 ± 8.7	66.29 ± 8.82	65.72 ± 9.37	<.0001	57.6 ± 8.07	55.95 ± 6.54	68.56 ± 8.71	66.31 ± 8.83	65.77 ± 9.41	<.0001
BMI	23.22 ± 3.06	22.51 ± 2.37	27.78 ± 3.12	26.79 ± 3.33	26.59 ± 3.55	<.0001	23.2 ± 3.06	22.51 ± 2.37	27.79 ± 3.13	26.79 ± 3.34	26.59 ± 3.58	<.0001
Waist circumference	75.19 ± 7.74	73.46 ± 6.23	86.37 ± 7.18	85.35 ± 7.61	84.99 ± 7.9	<.0001	75.13 ± 7.72	73.43 ± 6.23	86.33 ± 7.19	85.34 ± 7.63	84.96 ± 7.92	<.0001
SBP	117.28 ± 14.35	115.98 ± 13.74	125.54 ± 15.31	127.22 ± 15.79	126.18 ± 15.55	<.0001	117.16 ± 14.29	115.89 ± 13.69	125.38 ± 15.28	127.18 ± 15.73	126.05 ± 15.57	<.0001
DBP	73.18 ± 9.96	72.33 ± 9.62	78.52 ± 10.37	80.35 ± 10.87	79.63 ± 10.59	<.0001	73.12 ± 9.95	72.29 ± 9.61	78.47 ± 10.39	80.35 ± 10.86	79.59 ± 10.65	<.0001
HDL-C	60 ± 30.07	60.58 ± 26.89	55.95 ± 45.6	62 ± 39.67	60.09 ± 36.64	<.0001	60.02 ± 30.11	60.59 ± 26.92	55.99 ± 46.02	61.94 ± 39.42	59.92 ± 34.53	<.0001
LDL-C	113.15 ± 35.63	112.2 ± 34.37	119.92 ± 42.27	108.51 ± 39.42	108.1 ± 47.18	<.0001	112.99 ± 35.56	112.06 ± 34.29	119.75 ± 42.35	108.39 ± 39.43	108.2 ± 47.4	<.0001
Fasting glucose	93.67 ± 17.85	92.24 ± 15.24	102.75 ± 27.91	103.43 ± 26.31	105.04 ± 28.7	<.0001	93.59 ± 17.77	92.19 ± 15.18	102.65 ± 27.93	103.36 ± 26.28	104.94 ± 28.73	<.0001
Total cholesterol	192.05 ± 34	189.61 ± 32.69	207.84 ± 37.83	206.26 ± 36.78	205.12 ± 40.21	<.0001	191.84 ± 33.88	189.45 ± 32.6	207.59 ± 37.7	206.16 ± 36.76	205.25 ± 40.23	<.0001
eGFR	91.62 ± 18.18	91.83 ± 18.03	90.16 ± 19.13	92.19 ± 18.1	91.32 ± 19.28	<.0001	91.77 ± 18.14	91.95 ± 17.99	90.52 ± 19.06	92.3 ± 18.02	91.62 ± 19.16	<.0001
FLI	14.7 ± 16.14	9.34 ± 7	48.84 ± 15.75	52.18 ± 17.35	54.34 ± 17.63	<.0001	14.57 ± 16.04	9.3 ± 6.98	48.82 ± 15.76	52.16 ± 17.35	54.32 ± 17.65	<.0001
Triglyceride	101.99 ± 65.7	89.31 ± 44.49	182.73 ± 107.37	191.61 ± 115.46	199.29 ± 118.85	<.0001	101.6 ± 65.42	89.11 ± 44.35	182.66 ± 107.68	191.68 ± 115.6	199.14 ± 119.27	<.0001
Triglyceride	88.66<br>(88.56, 88.75)	80.78<br>(80.7, 80.86)	160.64<br>(160.18, 161.1)	167.19<br>(164.77, 169.64)	173.61<br>(169.77, 177.53)	<.0001	88.35<br>(88.26, 88.45)	80.62<br>(80.54, 80.7)	160.49<br>(160.02, 160.96)	167.21<br>(164.78, 169.68)	173.29<br>(169.4, 177.27)	<.0001
r-GTP	20.65 ± 22.04	17.55 ± 11.43	38.67 ± 42.31	65.44 ± 82.75	87.55 ± 128.04	<.0001	20.6 ± 22	17.53 ± 11.41	38.73 ± 42.55	65.45 ± 82.91	87.99 ± 128.96	<.0001
r-GTP*	17.18<br>(17.16, 17.2)	15.66<br>(15.65, 15.68)	30.26<br>(30.15, 30.38)	46.83<br>(45.86, 47.81)	54.65<br>(52.62, 56.76)	<.0001	17.14<br>(17.12, 17.16)	15.65<br>(15.63, 15.66)	30.28<br>(30.16, 30.39)	46.82<br>(45.84, 47.81)	54.88<br>(52.82, 57.02)	<.0001
ALT	18.92 ± 15.45	17.28 ± 12.06	29.37 ± 26.47	28.57 ± 25.24	34.48 ± 37.25	<.0001	18.86 ± 15.41	17.25 ± 12.04	29.37 ± 26.5	28.56 ± 25.29	34.55 ± 37.58	<.0001
ALT*	16.62<br>(16.6, 16.63)	15.63<br>(15.62, 15.64)	24.56<br>(24.48, 24.64)	24.07<br>(23.71, 24.44)	27.45<br>(26.74, 28.18)	<.0001	16.57<br>(16.56, 16.59)	15.6<br>(15.59, 15.62)	24.54<br>(24.47, 24.63)	24.07<br>(23.7, 24.44)	27.45<br>(26.72, 28.19)	<.0001
AST	21.61 ± 11.59	20.82 ± 9.42	26.45 ± 18.99	29.37 ± 27.94	35.01 ± 37.43	<.0001	21.56 ± 11.55	20.78 ± 9.4	26.39 ± 19	29.35 ± 27.99	35.05 ± 37.77	<.0001
AST*	20.39<br>(20.38, 20.41)	19.88<br>(19.86, 19.89)	23.92<br>(23.86, 23.98)	25.53<br>(25.2, 25.86)	28.64<br>(27.98, 29.32)	<.0001	20.35<br>(20.34, 20.37)	19.85<br>(19.83, 19.86)	23.86<br>(23.8, 23.92)	25.51<br>(25.18, 25.84)	28.62<br>(27.95, 29.31)	<.0001
CCI Score	0.77 ± 1.11	0.73 ± 1.07	0.99 ± 1.3	0.98 ± 1.24	1.84 ± 1.64	<.0001	0.76 ± 1.1	0.73 ± 1.07	0.97 ± 1.28	0.98 ± 1.24	1.82 ± 1.63	<.0001
HIS	32.18 ± 4.65	31.17 ± 3.72	38.7 ± 4.88	36.95 ± 4.86	37.07 ± 5.03	<.0001	32.15 ± 4.65	31.16 ± 3.72	38.73 ± 4.89	36.96 ± 4.87	37.07 ± 5.06	<.0001

* Geometric Mean (95% CI)

Cancer Type	SLD Group	**In Premenopause**							**In Premenopause (follow up until age 55)**						
		N	Event	Duration, PY	IR, 1000 PY	Model 1	Model 2	Model 3	N	Event	Duration, PY	IR, 1000 PY	Model 1	Model 2	Model 3
Cervical	No SLD	753210	2240	9237032.48	0.24	1 (ref.)	1 (ref.)	1 (ref.)	745610	1703	6783734.56	0.25	1 (ref.)	1 (ref.)	1 (ref.)
	MASLD	110782	376	1355047.71	0.28	1.144 (1.026, 1.276)	1.144 (1.025, 1.277)	**1.129 (1.011, 1.261)**	107398	239	887567.74	0.27	1.07 (0.934, 1.225)	1.074 (0.938, 1.23)	1.063 (0.928, 1.218)
	MetALD	4616	28	56187.35	0.50	2.056 (1.417, 2.985)	2.056 (1.417, 2.984)	**1.873 (1.285, 2.731)**	4571	20	38873.29	0.51	2.047 (1.317, 3.18)	2.054 (1.322, 3.192)	**1.832 (1.173, 2.863)**
	ALD	2016	8	24369.68	0.33	1.353 (0.676, 2.709)	1.353 (0.676, 2.709)	1.242 (0.619, 2.492)	1969	4	16175.08	0.25	0.982 (0.368, 2.619)	0.987 (0.37, 2.633)	0.887 (0.332, 2.371)
	p-value					0.0002	0.0002	0.0019					0.0124	0.0113	0.0514
Corpus	No SLD	753210	2474	9238231.51	0.27	1 (ref.)	1 (ref.)	1 (ref.)	745610	1620	6786203.85	0.24	1 (ref.)	1 (ref.)	1 (ref.)
	MASLD	110782	605	1354056.38	0.45	1.669 (1.527, 1.824)	1.611 (1.473, 1.762)	**1.636 (1.496, 1.79)**	107398	357	887239.04	0.40	1.727 (1.54, 1.936)	1.675 (1.493, 1.879)	**1.7 (1.515, 1.907)**
	MetALD	4616	15	56294.31	0.27	0.996 (0.599, 1.654)	0.98 (0.59, 1.628)	1.12 (0.673, 1.865)	4571	12	38926.69	0.31	1.319 (0.748, 2.328)	1.282 (0.726, 2.262)	1.464 (0.827, 2.592)
	ALD	2016	10	24389.65	0.41	1.534 (0.824, 2.855)	1.489 (0.8, 2.77)	1.67 (0.896, 3.113)	1969	2	16189.39	0.12	0.533 (0.133, 2.134)	0.512 (0.128, 2.048)	0.577 (0.144, 2.313)
	p-value					<.0001	<.0001	<.0001					<.0001	<.0001	<.0001
Ovarian	No SLD	753210	3704	9234548.06	0.40	1 (ref.)	1 (ref.)	1 (ref.)	745610	2495	6783163.77	0.37	1 (ref.)	1 (ref.)	1 (ref.)
	MASLD	110782	663	1354322.13	0.49	1.22 (1.123, 1.325)	1.218 (1.121, 1.323)	**1.22 (1.122, 1.326)**	107398	389	887291.02	0.44	1.227 (1.103, 1.366)	1.219 (1.095, 1.356)	**1.22 (1.096, 1.359)**
	MetALD	4616	26	56245.90	0.46	1.152 (0.783, 1.695)	1.151 (0.783, 1.693)	1.166 (0.791, 1.719)	4571	14	38897.18	0.36	1.003 (0.593, 1.696)	0.996 (0.589, 1.685)	1.025 (0.604, 1.738)
	ALD	2016	11	24356.21	0.45	1.123 (0.622, 2.03)	1.121 (0.62, 2.027)	1.128 (0.623, 2.041)	1969	4	16191.67	0.25	0.696 (0.261, 1.854)	0.689 (0.259, 1.836)	0.704 (0.264, 1.881)
	p-value					<.0001	<.0001	<.0001					0.0021	0.0033	0.0032

Supplementary Table S5. Sensitivity analysis

Outcome	SLD Group	FLI	In Pre-menopause				In Pre-menopause			
			IR per 1,000	Model 1	Model 2	Model 3	IR per 1,000	Model 1	Model 2	Model 3
Cervical cancer	No SLD	< 30	0.24	1(Ref.)	1(Ref.)	1(Ref.)	0.25	1(Ref.)	1(Ref.)	1(Ref.)
		30 - 59	0.27	1.107 (0.981, 1.249)	1.106 (0.980, 1.249)	1.089 (0.964, 1.230)	0.28	1.120 (1.039, 1.206)	1.100 (1.020, 1.185)	**1.081 (1.002, 1.165)**
	MASLD	≥ 60	0.31	1.286 (1.049, 1.578)	1.286 (1.048, 1.578)	**1.264 (1.030, 1.551)**	0.33	1.321 (1.178, 1.482)	1.298 (1.156, 1.456)	**1.264 (1.126, 1.419)**
	MetALD		0.76	3.126 (1.813, 5.391)	3.126 (1.812, 5.390)	**2.830 (1.636, 4.896)**	0.59	2.359 (1.269, 4.385)	2.430 (1.307, 4.519)	**2.220 (1.193, 4.135)**
	ALD		0.58	2.398 (0.997, 5.766)	2.397 (0.997, 5.765)	2.198 (0.912, 5.295)	0.30	1.183 (0.492, 2.843)	1.189 (0.495, 2.860)	1.115 (0.464, 2.682)
	p-value			<.0001	<.0001	0.0002		<.0001	<.0001	0.0001
Endometrial cancer	No SLD	< 30	0.27	1(Ref.)	1(Ref.)	1(Ref.)	0.18	1(Ref.)	1(Ref.)	1(Ref.)
		30 - 59	0.39	1.466 (1.324, 1.623)	1.414 (1.277, 1.566)	**1.445 (1.304, 1.601)**	0.22	1.214 (1.115, 1.323)	1.298 (1.191, 1.415)	**1.316 (1.206, 1.435)**
	MASLD	≥ 60	0.62	2.314 (1.996, 2.681)	2.240 (1.932, 2.597)	**2.273 (1.960, 2.636)**	0.28	1.591 (1.404, 1.804)	1.701 (1.499, 1.929)	**1.745 (1.537, 1.980)**
	MetALD		0.35	1.304 (0.585, 2.906)	1.282 (0.576, 2.857)	1.485 (0.665, 3.314)	0.18	0.985 (0.317, 3.056)	0.900 (0.290, 2.795)	0.971 (0.312, 3.015)
	ALD		0.23	0.868 (0.217, 3.474)	0.842 (0.211, 3.370)	0.942 (0.235, 3.771)	0.24	1.327 (0.497, 3.539)	1.310 (0.491, 3.495)	1.363 (0.511, 3.637)
	p-value			<.0001	<.0001	<.0001		<.0001	<.0001	<.0001
Ovarian cancer	No SLD	< 30	0.40	1(Ref.)	1(Ref.)	1(Ref.)	0.39	1(Ref.)	1(Ref.)	1(Ref.)
		30 - 59	0.45	1.125 (1.025, 1.235)	1.123 (1.023, 1.233)	**1.128 (1.027, 1.239)**	0.42	1.071 (1.008, 1.138)	1.062 (0.999, 1.129)	**1.071 (1.007, 1.138)**
	MASLD	≥ 60	0.61	1.534 (1.325, 1.775)	1.531 (1.322, 1.772)	**1.522 (1.314, 1.762)**	0.52	1.349 (1.231, 1.479)	1.338 (1.221, 1.467)	**1.351 (1.232, 1.482)**
	MetALD		0.70	1.742 (0.988, 3.070)	1.740 (0.987, 3.067)	1.757 (0.995, 3.103)	0.65	1.680 (0.932, 3.029)	1.703 (0.944, 3.070)	1.657 (0.916, 2.998)
	ALD		0.23	0.577 (0.144, 2.309)	0.576 (0.144, 2.304)	0.576 (0.144, 2.306)	0.30	0.767 (0.319, 1.844)	0.769 (0.320, 1.849)	0.760 (0.316, 1.829)
	p-value			<.0001	<.0001	<.0001		<.0001	<.0001	<.0001

Supplementary Table S6. Forest plots of subgroup analyses by menopausal status and cancer type.

1) Cervical Cancer in Pre-menopause

Subgroup	SLD group	N	Event	IR per 1,000	Model 3	p for inter
Smoking, Non-Ex	No SLD	730463	2161	0.24	1 (ref.)	0.5473
	MASLD	106315	355	0.27	1.119 (0.999, 1.253)	
	MetALD	3318	21	0.52	2.028 (1.318, 3.119)	
	ALD	1466	7	0.39	1.555 (0.74, 3.267)	
Smoking, Current	No SLD	22747	79	0.28	1 (ref.)	
	MASLD	4467	21	0.39	1.344 (0.831, 2.175)	
	MetALD	1298	7	0.45	1.532 (0.707, 3.32)	
	ALD	550	1	0.15	0.518 (0.072, 3.723)	
Regular exercise, No	No SLD	621160	1843	0.24	1 (ref.)	0.5735
	MASLD	93990	326	0.28	1.158 (1.028, 1.304)	
	MetALD	3808	25	0.54	2.033 (1.364, 3.03)	
	ALD	1682	8	0.39	1.492 (0.743, 2.996)	
Regular exercise, Yes	No SLD	132050	397	0.24	1 (ref.)	
	MASLD	16792	50	0.24	0.977 (0.728, 1.311)	
	MetALD	808	3	0.3	1.127 (0.362, 3.515)	
	ALD	334	0	0	-	
Age at menarche ≤ 12	No SLD	33077	89	0.22	1 (ref.)	0.7245
	MASLD	5948	13	0.18	0.81 (0.453, 1.45)	
	MetALD	185	1	0.45	1.818 (0.253, 13.069)	
	ALD	86	0	0	-	
Age at menarche > 12	No SLD	720133	2151	0.24	1 (ref.)	
	MASLD	104834	363	0.28	1.145 (1.023, 1.281)	
	MetALD	4431	27	0.5	1.874 (1.277, 2.751)	
	ALD	1930	8	0.34	1.29 (0.643, 2.589)	
OC, <1 years	No SLD	729000	2161	0.24	1 (ref.)	0.7908
	MASLD	106021	355	0.27	1.119 (0.999, 1.253)	
	MetALD	4143	26	0.52	1.954 (1.322, 2.888)	
	ALD	1819	8	0.36	1.393 (0.694, 2.794)	
OC, ≥1 year	No SLD	24210	79	0.27	1 (ref.)	
	MASLD	4761	21	0.36	1.348 (0.833, 2.182)	
	MetALD	473	2	0.35	1.215 (0.298, 4.951)	
	ALD	197	0	0	-	
Parity, No	No SLD	27851	78	0.23	1 (ref.)	0.5134
	MASLD	3446	11	0.26	1.144 (0.608, 2.151)	
	MetALD	218	3	1.15	4.472 (1.408, 14.209)	
	ALD	118	0	0	-	
Parity, Yes	No SLD	725359	2162	0.24	1 (ref.)	
	MASLD	107336	365	0.28	1.129 (1.009, 1.262)	
	MetALD	4398	25	0.47	1.75 (1.175, 2.607)	
	ALD	1898	8	0.35	1.32 (0.658, 2.648)	
Breast Feeding, No	No SLD	135475	389	0.23	1 (ref.)	0.8665
	MASLD	18713	67	0.29	1.247 (0.962, 1.617)	
	MetALD	934	6	0.53	2.033 (0.905, 4.565)	
	ALD	454	0	0	-	
Breast Feeding, Yes	No SLD	617735	1851	0.24	1 (ref.)	
	MASLD	92069	309	0.27	1.106 (0.98, 1.249)	
	MetALD	3682	22	0.49	1.831 (1.198, 2.798)	
	ALD	1562	8	0.42	1.596 (0.796, 3.202)	

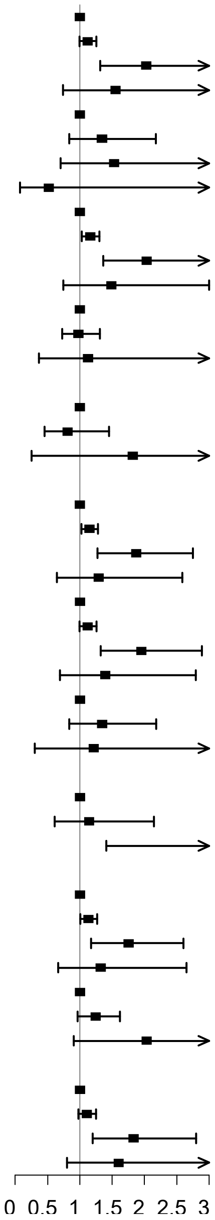

2) Endometrial Cancer in Pre-menopause

Subgroup	SLD group	N	Event	IR per 1,000	Model 3	p for inter
Smoking, Non-Ex	No SLD	730463	2421	0.27	1 (ref.)	0.6526
	MASLD	106315	593	0.46	1.65 (1.506, 1.807)	
	MetALD	3318	11	0.27	1.03 (0.569, 1.863)	
	ALD	1466	8	0.45	1.663 (0.83, 3.329)	
Smoking, Current	No SLD	22747	53	0.19	1 (ref.)	
	MASLD	4467	12	0.22	1.151 (0.615, 2.154)	
	MetALD	1298	4	0.25	1.397 (0.506, 3.861)	
	ALD	550	2	0.3	1.612 (0.393, 6.614)	
Regular exercise, No	No SLD	621160	1989	0.26	1 (ref.)	0.1411
	MASLD	93990	524	0.46	1.708 (1.55, 1.883)	
	MetALD	3808	11	0.24	1.025 (0.565, 1.857)	
	ALD	1682	9	0.44	1.841 (0.955, 3.549)	
Regular exercise, Yes	No SLD	132050	485	0.3	1 (ref.)	
	MASLD	16792	81	0.39	1.296 (1.024, 1.64)	
	MetALD	808	4	0.4	1.504 (0.562, 4.026)	
	ALD	334	1	0.25	0.914 (0.128, 6.501)	
Age at menarche ≤ 12	No SLD	33077	121	0.3	1 (ref.)	0.8825
	MASLD	5948	38	0.52	1.675 (1.163, 2.413)	
	MetALD	185	0	0	-	
	ALD	86	1	1.01	3.664 (0.512, 26.234)	
Age at menarche > 12	No SLD	720133	2353	0.27	1 (ref.)	
	MASLD	104834	567	0.44	1.634 (1.489, 1.793)	
	MetALD	4431	15	0.28	1.17 (0.703, 1.949)	
	ALD	1930	9	0.38	1.575 (0.817, 3.035)	
OC, <1 years	No SLD	729000	2420	0.27	1 (ref.)	0.9583
	MASLD	106021	589	0.45	1.64 (1.497, 1.796)	
	MetALD	4143	14	0.28	1.122 (0.662, 1.901)	
	ALD	1819	9	0.41	1.606 (0.834, 3.095)	
OC, ≥1 year	No SLD	24210	54	0.18	1 (ref.)	
	MASLD	4761	16	0.27	1.503 (0.86, 2.626)	
	MetALD	473	1	0.17	1.082 (0.149, 7.824)	
	ALD	197	1	0.42	2.583 (0.357, 18.694)	
Parity, No	No SLD	27851	133	0.39	1 (ref.)	<.0001
	MASLD	3446	57	1.37	3.45 (2.529, 4.706)	
	MetALD	218	1	0.38	1.199 (0.167, 8.586)	
	ALD	118	0	0	-	
Parity, Yes	No SLD	725359	2341	0.26	1 (ref.)	
	MASLD	107336	548	0.42	1.548 (1.409, 1.7)	
	MetALD	4398	14	0.26	1.116 (0.659, 1.891)	
	ALD	1898	10	0.44	1.808 (0.97, 3.37)	
Breast Feeding, No	No SLD	135475	489	0.29	1 (ref.)	<.0001
	MASLD	18713	167	0.73	2.489 (2.087, 2.968)	
	MetALD	934	4	0.35	1.4 (0.522, 3.751)	
	ALD	454	1	0.18	0.721 (0.101, 5.137)	
Breast Feeding, Yes	No SLD	617735	1985	0.26	1 (ref.)	
	MASLD	92069	438	0.39	1.441 (1.298, 1.6)	
	MetALD	3682	11	0.24	1.045 (0.577, 1.893)	
	ALD	1562	9	0.48	1.962 (1.018, 3.78)	

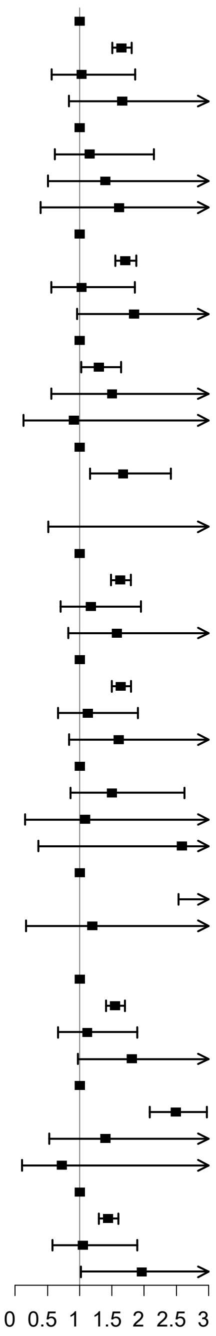

3) Ovarian Cancer in Pre-menopause

Subgroup	SLD group	N	Event	IR per 1,000	Model 3	p for inter
Smoking, Non-Ex	No SLD	730463	3594	0.4	1 (ref.)	0.5282
	MASLD	106315	633	0.49	1.212 (1.113, 1.32)	
	MetALD	3318	18	0.44	1.114 (0.701, 1.771)	
	ALD	1466	10	0.56	1.406 (0.756, 2.616)	
Smoking, Current	No SLD	22747	110	0.4	1 (ref.)	
	MASLD	4467	30	0.55	1.409 (0.941, 2.11)	
	MetALD	1298	8	0.51	1.33 (0.649, 2.726)	
	ALD	550	1	0.15	0.387 (0.054, 2.764)	
Regular exercise, No	No SLD	621160	3071	0.4	1 (ref.)	0.83
	MASLD	93990	572	0.5	1.235 (1.129, 1.351)	
	MetALD	3808	22	0.47	1.191 (0.781, 1.816)	
	ALD	1682	10	0.49	1.22 (0.655, 2.273)	
Regular exercise, Yes	No SLD	132050	633	0.39	1 (ref.)	
	MASLD	16792	91	0.44	1.136 (0.912, 1.416)	
	MetALD	808	4	0.4	1.046 (0.391, 2.799)	
	ALD	334	1	0.25	0.643 (0.09, 4.573)	
Age at menarche ≤ 12	No SLD	33077	206	0.51	1 (ref.)	0.998
	MASLD	5948	45	0.62	1.204 (0.872, 1.663)	
	MetALD	185	0	0	-	
	ALD	86	0	0	-	
Age at menarche > 12	No SLD	720133	3498	0.4	1 (ref.)	
	MASLD	104834	618	0.48	1.221 (1.12, 1.331)	
	MetALD	4431	26	0.48	1.229 (0.834, 1.812)	
	ALD	1930	11	0.47	1.189 (0.657, 2.153)	
OC, <1 years	No SLD	729000	3594	0.4	1 (ref.)	0.8912
	MASLD	106021	641	0.49	1.227 (1.127, 1.336)	
	MetALD	4143	24	0.48	1.187 (0.793, 1.778)	
	ALD	1819	11	0.5	1.242 (0.686, 2.248)	
OC, ≥1 year	No SLD	24210	110	0.37	1 (ref.)	
	MASLD	4761	22	0.38	1.034 (0.654, 1.635)	
	MetALD	473	2	0.35	0.933 (0.23, 3.783)	
	ALD	197	0	0	-	
Parity, No	No SLD	27851	204	0.6	1 (ref.)	0.6795
	MASLD	3446	38	0.91	1.504 (1.063, 2.126)	
	MetALD	218	0	0	-	
	ALD	118	0	0	-	
Parity, Yes	No SLD	725359	3500	0.39	1 (ref.)	
	MASLD	107336	625	0.48	1.206 (1.106, 1.314)	
	MetALD	4398	26	0.48	1.251 (0.849, 1.844)	
	ALD	1898	11	0.48	1.23 (0.68, 2.227)	
Breast Feeding, No	No SLD	135475	777	0.47	1 (ref.)	0.0402
	MASLD	18713	151	0.66	1.414 (1.188, 1.684)	
	MetALD	934	2	0.18	0.38 (0.095, 1.524)	
	ALD	454	1	0.18	0.39 (0.055, 2.776)	
Breast Feeding, Yes	No SLD	617735	2927	0.39	1 (ref.)	
	MASLD	92069	512	0.45	1.172 (1.066, 1.288)	
	MetALD	3682	24	0.53	1.408 (0.94, 2.108)	
	ALD	1562	10	0.53	1.39 (0.746, 2.589)	

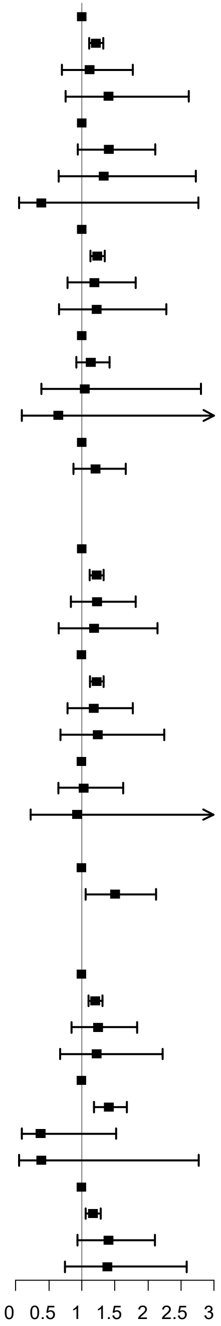

4) Cervical Cancer in Post-menopause

Subgroup	SLD group	N	Event	IR per 1,000	Model 3	p for inter
Smoking, Non-Ex	No SLD	824188	2449	0.25	1 (ref.)	0.309
	MASLD	353950	1204	0.29	1.112 (1.038, 1.193)	
	MetALD	3391	14	0.34	1.369 (0.809, 2.317)	
	ALD	3797	10	0.23	0.878 (0.472, 1.635)	
Smoking, Current	No SLD	20786	72	0.3	1 (ref.)	
	MASLD	10147	50	0.43	1.375 (0.958, 1.972)	
	MetALD	922	5	0.45	1.537 (0.621, 3.806)	
	ALD	493	4	0.72	2.382 (0.87, 6.519)	
Regular exercise, No	No SLD	683193	2056	0.25	1 (ref.)	0.6838
	MASLD	305154	1045	0.29	1.106 (1.026, 1.193)	
	MetALD	3479	17	0.41	1.51 (0.935, 2.44)	
	ALD	3596	12	0.29	1.075 (0.609, 1.896)	
Regular exercise, Yes	No SLD	161781	465	0.24	1 (ref.)	
	MASLD	58943	209	0.3	1.193 (1.013, 1.405)	
	MetALD	834	2	0.2	0.794 (0.198, 3.186)	
	ALD	694	2	0.24	0.981 (0.245, 3.935)	
Age at menarche ≤ 12	No SLD	8459	33	0.32	1 (ref.)	0.9859
	MASLD	3156	13	0.34	0.998 (0.525, 1.897)	
	MetALD	58	0	0	-	
	ALD	60	0	0	-	
Age at menarche > 12	No SLD	836515	2488	0.25	1 (ref.)	
	MASLD	360941	1241	0.29	1.122 (1.047, 1.202)	
	MetALD	4255	19	0.37	1.405 (0.893, 2.211)	
	ALD	4230	14	0.28	1.081 (0.639, 1.829)	
OC, <1 years	No SLD	796012	2353	0.25	1 (ref.)	0.3142
	MASLD	338956	1170	0.29	1.135 (1.057, 1.218)	
	MetALD	3871	15	0.32	1.23 (0.739, 2.047)	
	ALD	3948	14	0.3	1.167 (0.69, 1.974)	
OC, ≥1 year	No SLD	48962	168	0.28	1 (ref.)	
	MASLD	25141	84	0.28	0.946 (0.727, 1.229)	
	MetALD	442	4	0.75	2.47 (0.915, 6.667)	
	ALD	342	0	0	-	
Parity, No	No SLD	15267	38	0.21	1 (ref.)	0.6969
	MASLD	4686	13	0.23	1.089 (0.58, 2.044)	
	MetALD	130	0	0	-	
	ALD	110	1	0.8	3.466 (0.475, 25.268)	
Parity, Yes	No SLD	829707	2483	0.25	1 (ref.)	
	MASLD	359411	1241	0.29	1.121 (1.046, 1.201)	
	MetALD	4183	19	0.38	1.417 (0.901, 2.231)	
	ALD	4180	13	0.27	1.007 (0.584, 1.737)	
Breast Feeding, No	No SLD	58301	141	0.2	1 (ref.)	0.9898
	MASLD	18247	53	0.24	1.169 (0.852, 1.604)	
	MetALD	444	0	0	-	
	ALD	330	1	0.26	1.187 (0.166, 8.493)	
Breast Feeding, Yes	No SLD	786673	2380	0.25	1 (ref.)	
	MASLD	345850	1201	0.29	1.118 (1.043, 1.2)	
	MetALD	3869	19	0.41	1.514 (0.962, 2.382)	
	ALD	3960	13	0.28	1.052 (0.61, 1.815)	
Age at menopause < 40	No SLD	13915	51	0.32	1 (ref.)	0.2121
	MASLD	7268	17	0.2	0.623 (0.36, 1.079)	
	MetALD	92	0	0	-	
	ALD	87	0	0	-	
Age at menopause ≥ 40	No SLD	831059	2470	0.25	1 (ref.)	
	MASLD	356829	1237	0.29	1.132 (1.057, 1.213)	
	MetALD	4221	19	0.37	1.413 (0.898, 2.225)	
	ALD	4203	14	0.29	1.086 (0.642, 1.838)	
HRT < 2 years	No SLD	781409	2373	0.26	1 (ref.)	0.2882
	MASLD	347366	1194	0.29	1.105 (1.03, 1.185)	
	MetALD	3994	19	0.4	1.465 (0.93, 2.305)	
	ALD	4023	14	0.3	1.115 (0.659, 1.887)	
HRT ≥ 2 years	No SLD	63565	148	0.19	1 (ref.)	
	MASLD	16731	60	0.3	1.497 (1.109, 2.021)	
	MetALD	319	0	0	-	
	ALD	267	0	0	-	

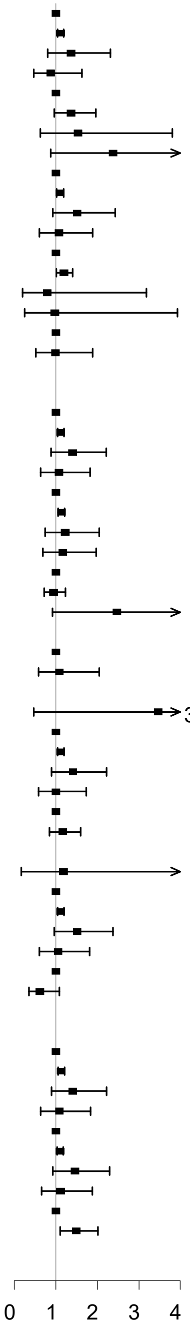

5) Endometrial Cancer in Post-menopause

Subgroup	SLD group	N	Event	IR per 1,000	Model 3	p for inter
Smoking, Non-Ex	No SLD	824188	1773	0.18	1 (ref.)	0.9334
	MASLD	353950	985	0.24	1.42 (1.311, 1.537)	
	MetALD	3391	10	0.24	1.243 (0.667, 2.315)	
	ALD	3797	6	0.14	0.77 (0.345, 1.716)	
Smoking, Current	No SLD	20786	30	0.13	1 (ref.)	
	MASLD	10147	22	0.19	1.628 (0.939, 2.823)	
	MetALD	922	0	0	-	
	ALD	493	1	0.18	1.292 (0.176, 9.474)	
Regular exercise, No	No SLD	683193	1397	0.17	1 (ref.)	0.9291
	MASLD	305154	817	0.23	1.425 (1.306, 1.555)	
	MetALD	3479	8	0.19	1.068 (0.532, 2.143)	
	ALD	3596	5	0.12	0.718 (0.298, 1.728)	
Regular exercise, Yes	No SLD	161781	406	0.21	1 (ref.)	
	MASLD	58943	190	0.27	1.417 (1.192, 1.684)	
	MetALD	834	2	0.2	0.927 (0.231, 3.723)	
	ALD	694	2	0.24	1.231 (0.307, 4.941)	
Age at menarche ≤ 12	No SLD	8459	24	0.23	1 (ref.)	0.2615
	MASLD	3156	9	0.24	1.114 (0.518, 2.398)	
	MetALD	58	1	1.41	6.663 (0.909, 48.831)	
	ALD	60	0	0	-	
Age at menarche > 12	No SLD	836515	1779	0.18	1 (ref.)	
	MASLD	360941	998	0.23	1.427 (1.319, 1.544)	
	MetALD	4255	9	0.18	0.948 (0.491, 1.827)	
	ALD	4230	7	0.14	0.828 (0.394, 1.74)	
OC, <1 years	No SLD	796012	1693	0.18	1 (ref.)	0.8993
	MASLD	338956	939	0.23	1.431 (1.319, 1.551)	
	MetALD	3871	10	0.21	1.164 (0.624, 2.172)	
	ALD	3948	6	0.13	0.762 (0.342, 1.7)	
OC, ≥1 year	No SLD	48962	110	0.19	1 (ref.)	
	MASLD	25141	68	0.23	1.324 (0.978, 1.793)	
	MetALD	442	0	0	-	
	ALD	342	1	0.25	1.379 (0.192, 9.877)	
Parity, No	No SLD	15267	59	0.32	1 (ref.)	0.9696
	MASLD	4686	26	0.47	1.593 (1.004, 2.528)	
	MetALD	130	0	0	-	
	ALD	110	0	0	-	
Parity, Yes	No SLD	829707	1744	0.18	1 (ref.)	
	MASLD	359411	981	0.23	1.419 (1.311, 1.537)	
	MetALD	4183	10	0.2	1.085 (0.582, 2.024)	
	ALD	4180	7	0.14	0.852 (0.405, 1.79)	
Breast Feeding, No	No SLD	58301	183	0.26	1 (ref.)	0.9579
	MASLD	18247	80	0.37	1.526 (1.173, 1.985)	
	MetALD	444	0	0	-	
	ALD	330	0	0	-	
Breast Feeding, Yes	No SLD	786673	1620	0.17	1 (ref.)	
	MASLD	345850	927	0.23	1.415 (1.303, 1.535)	
	MetALD	3869	10	0.21	1.192 (0.639, 2.223)	
	ALD	3960	7	0.15	0.915 (0.435, 1.923)	
Age at menopause < 40	No SLD	13915	15	0.09	1 (ref.)	0.9964
	MASLD	7268	9	0.11	1.305 (0.571, 2.984)	
	MetALD	92	0	0	-	
	ALD	87	0	0	-	
Age at menopause ≥ 40	No SLD	831059	1788	0.18	1 (ref.)	
	MASLD	356829	998	0.24	1.425 (1.317, 1.541)	
	MetALD	4221	10	0.2	1.05 (0.563, 1.958)	
	ALD	4203	7	0.14	0.824 (0.392, 1.732)	
HRT < 2 years	No SLD	781409	1601	0.17	1 (ref.)	0.0111
	MASLD	347366	966	0.24	1.477 (1.362, 1.601)	
	MetALD	3994	9	0.19	1.043 (0.541, 2.012)	
	ALD	4023	6	0.13	0.777 (0.348, 1.733)	
HRT ≥ 2 years	No SLD	63565	202	0.26	1 (ref.)	
	MASLD	16731	41	0.2	0.826 (0.59, 1.156)	
	MetALD	319	1	0.26	0.983 (0.138, 7.015)	
	ALD	267	1	0.31	1.169 (0.164, 8.343)	

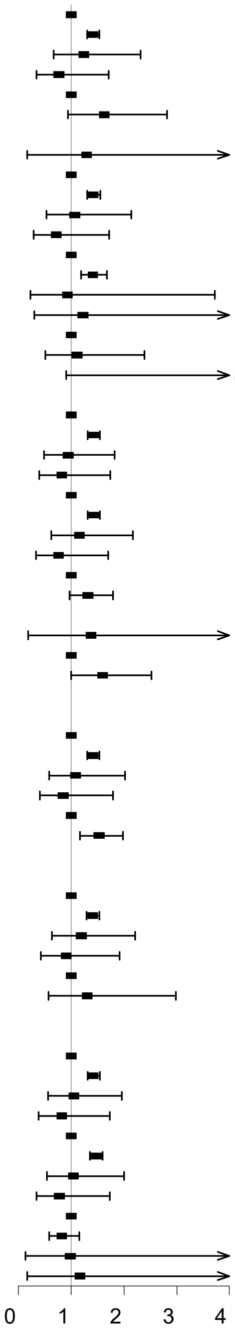

Subgroup	SLD group	N	Event	IR per 1,000	Model 3	p for inter
Smoking, Non-Ex	No SLD	824188	3800	0.39	1 (ref.)	0.6516
	MASLD	353950	1840	0.44	1.139 (1.077, 1.205)	
	MetALD	3391	13	0.32	0.832 (0.483, 1.435)	
	ALD	3797	16	0.36	0.937 (0.573, 1.531)	
Smoking, Current	No SLD	20786	109	0.46	1 (ref.)	
	MASLD	10147	55	0.48	1.046 (0.756, 1.447)	
	MetALD	922	7	0.63	1.399 (0.651, 3.004)	
	ALD	493	0	0	-	
Regular exercise, No	No SLD	683193	3139	0.39	1 (ref.)	0.9217
	MASLD	305154	1570	0.44	1.128 (1.061, 1.2)	
	MetALD	3479	16	0.38	0.969 (0.592, 1.586)	
	ALD	3596	14	0.33	0.857 (0.507, 1.449)	
Regular exercise, Yes	No SLD	161781	770	0.39	1 (ref.)	
	MASLD	58943	325	0.46	1.173 (1.03, 1.336)	
	MetALD	834	4	0.4	1.001 (0.374, 2.674)	
	ALD	694	2	0.24	0.617 (0.154, 2.471)	
Age at menarche ≤ 12	No SLD	8459	51	0.5	1 (ref.)	0.9757
	MASLD	3156	19	0.5	1.013 (0.598, 1.715)	
	MetALD	58	0	0	-	
	ALD	60	0	0	-	
Age at menarche > 12	No SLD	836515	3858	0.39	1 (ref.)	
	MASLD	360941	1876	0.44	1.138 (1.076, 1.203)	
	MetALD	4255	20	0.39	0.992 (0.638, 1.542)	
	ALD	4230	16	0.32	0.833 (0.51, 1.361)	
OC, <1 years	No SLD	796012	3697	0.39	1 (ref.)	0.0994
	MASLD	338956	1765	0.44	1.131 (1.068, 1.198)	
	MetALD	3871	18	0.39	0.973 (0.612, 1.549)	
	ALD	3948	12	0.26	0.663 (0.376, 1.169)	
OC, ≥1 year	No SLD	48962	212	0.36	1 (ref.)	
	MASLD	25141	130	0.43	1.22 (0.98, 1.518)	
	MetALD	442	2	0.37	1.01 (0.251, 4.068)	
	ALD	342	4	1	2.757 (1.029, 7.384)	
Parity, No	No SLD	15267	121	0.66	1 (ref.)	0.5847
	MASLD	4686	32	0.58	0.866 (0.586, 1.278)	
	MetALD	130	0	0	-	
	ALD	110	0	0	-	
Parity, Yes	No SLD	829707	3788	0.38	1 (ref.)	
	MASLD	359411	1863	0.44	1.143 (1.081, 1.209)	
	MetALD	4183	20	0.4	1.026 (0.66, 1.594)	
	ALD	4180	16	0.33	0.853 (0.522, 1.395)	
Breast Feeding, No	No SLD	58301	331	0.47	1 (ref.)	0.9748
	MASLD	18247	120	0.55	1.161 (0.942, 1.43)	
	MetALD	444	2	0.37	0.765 (0.19, 3.075)	
	ALD	330	0	0	-	
Breast Feeding, Yes	No SLD	786673	3578	0.38	1 (ref.)	
	MASLD	345850	1775	0.43	1.135 (1.071, 1.202)	
	MetALD	3869	18	0.39	1.006 (0.632, 1.6)	
	ALD	3960	16	0.35	0.905 (0.553, 1.478)	
Age at menopause < 40	No SLD	13915	53	0.33	1 (ref.)	0.9426
	MASLD	7268	27	0.32	0.988 (0.622, 1.571)	
	MetALD	92	0	0	-	
	ALD	87	0	0	-	
Age at menopause ≥ 40	No SLD	831059	3856	0.39	1 (ref.)	
	MASLD	356829	1868	0.44	1.139 (1.077, 1.204)	
	MetALD	4221	20	0.39	0.993 (0.639, 1.543)	
	ALD	4203	16	0.33	0.831 (0.509, 1.359)	
HRT < 2 years	No SLD	781409	3548	0.38	1 (ref.)	0.1888
	MASLD	347366	1810	0.44	1.152 (1.088, 1.22)	
	MetALD	3994	19	0.4	1.018 (0.648, 1.6)	
	ALD	4023	14	0.3	0.776 (0.459, 1.313)	
HRT ≥ 2 years	No SLD	63565	361	0.47	1 (ref.)	
	MASLD	16731	85	0.42	0.903 (0.713, 1.143)	
	MetALD	319	1	0.26	0.544 (0.076, 3.872)	
	ALD	267	2	0.62	1.313 (0.327, 5.27)	

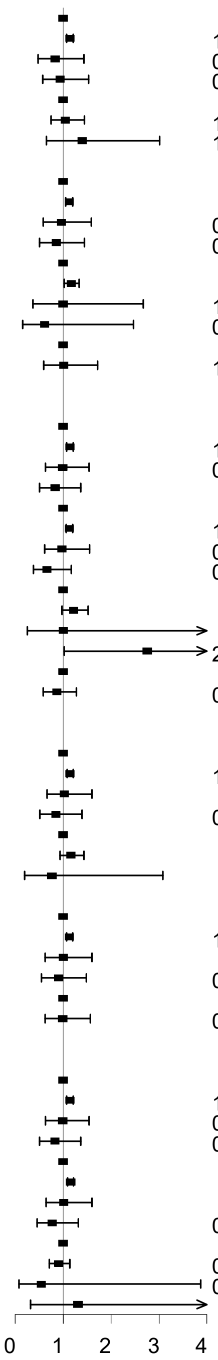

Supplement: Supplementary file 1 [file cancers-18-00894-s001.zip › cancers-4116609-supplementary.pdf]
